# Wnt/β-Catenin Signaling Promotes Differentiation of Ischemia-Activated Adult Neural Stem/Progenitor Cells to Neuronal Precursors

**DOI:** 10.3389/fnins.2021.628983

**Published:** 2021-02-25

**Authors:** Jan Kriska, Lucie Janeckova, Denisa Kirdajova, Pavel Honsa, Tomas Knotek, David Dzamba, Denisa Kolenicova, Olena Butenko, Martina Vojtechova, Martin Capek, Zbynek Kozmik, Makoto Mark Taketo, Vladimir Korinek, Miroslava Anderova

**Affiliations:** ^1^Department of Cellular Neurophysiology, Institute of Experimental Medicine, Czech Academy of Sciences, Prague, Czechia; ^2^Laboratory of Cell and Developmental Biology, Institute of Molecular Genetics, Czech Academy of Sciences, Prague, Czechia; ^3^Second Faculty of Medicine, Charles University, Prague, Czechia; ^4^Service Laboratory of Light Microscopy, Institute of Molecular Genetics, Czech Academy of Sciences, Prague, Czechia; ^5^Laboratory of Transcriptional Regulation, Institute of Molecular Genetics, Czech Academy of Sciences, Prague, Czechia; ^6^Division of Experimental Therapeutics, Graduate School of Medicine, Kyoto University, Kyoto, Japan

**Keywords:** Wnt signaling, transgenic mouse, neural stem/progenitor cell, focal cerebral ischemia, adult neurogenesis, gliogenesis, patch-clamp technique, single-cell RNA sequencing

## Abstract

Modulating endogenous regenerative processes may represent a suitable treatment for central nervous system (CNS) injuries, such as stroke or trauma. Neural stem/progenitor cells (NS/PCs), which naturally reside in the subventricular zone (SVZ) of the adult brain, proliferate and differentiate to other cell types, and therefore may compensate the negative consequences of ischemic injury. The fate of NS/PCs in the developing brain is largely influenced by Wingless/Integrated (Wnt) signaling; however, its role in the differentiation of adult NS/PCs under ischemic conditions is still enigmatic. In our previous study, we identified the Wnt/β-catenin signaling pathway as a factor promoting neurogenesis at the expense of gliogenesis in neonatal mice. In this study, we used adult transgenic mice in order to assess the impact of the canonical Wnt pathway modulation (inhibition or hyper-activation) on NS/PCs derived from the SVZ, and combined it with the middle cerebral artery occlusion (MCAO) to disclose the effect of focal cerebral ischemia (FCI). Based on the electrophysiological properties of cultured cells, we first identified three cell types that represented *in vitro* differentiated NS/PCs – astrocytes, neuron-like cells, and precursor cells. Following FCI, we detected fewer neuron-like cells after Wnt signaling inhibition. Furthermore, the immunohistochemical analysis revealed an overall higher expression of cell-type-specific proteins after FCI, indicating increased proliferation and differentiation rates of NS/PCs in the SVZ. Remarkably, Wnt signaling hyper-activation increased the abundance of proliferating and neuron-like cells, while Wnt pathway inhibition had the opposite effect. Finally, the expression profiling at the single cell level revealed an increased proportion of neural stem cells and neuroblasts after FCI. These observations indicate that Wnt signaling enhances NS/PCs-based regeneration in the adult mouse brain following FCI, and supports neuronal differentiation in the SVZ.

## Introduction

Stroke is one of the leading causes of mortality worldwide, affecting a great number of people in developed countries and thus imposing a considerable economic burden on society (Woodruff et al., 2011; Rajsic et al., 2019). Aging, genetic predisposition, and unhealthy lifestyle, are all among the risk factors for stroke (Boehme et al., 2017). Ischemic stroke due to a blocked artery comprises more than 85% of all stroke cases (Woodruff et al., 2011); the most frequently affected vessel of the brain is the middle cerebral artery. Its occlusion causes poor blood flow followed by glucose and oxygen deprivation, resulting in cell death in a large cortical area of the brain (Rossi et al., 2007; Puig et al., 2018; Belov Kirdajova et al., 2020). Nevertheless, FCI also has a consistent impact on the physiological functions of cells residing in regions distant from the lesion (Ginsberg, 2003; Wang et al., 2017). Ischemic brain injury leads to motoric, sensoric, and cognitive dysfunctions, and it is accompanied by elevated neurogenesis in the neurogenic niches of the postnatal brain. Therefore, recent studies have attempted to treat or replace diminishing numbers of neural cells with the use of stem cells from various sources (Ruzicka et al., 2017), or by utilizing NS/PCs naturally residing in the adult brain (Groves et al., 2019) with the capability of differentiating to other cell types (Kriska et al., 2016; Butti et al., 2019; Vancamp et al., 2019).

Adult neurogenesis in the CNS was first documented in the 1960s (Altman and Das, 1965). Under physiological conditions, it takes place in two particular regions of the mammalian brain – the SGZ of the dentate gyrus in the hippocampus and the SVZ of the lateral ventricles (LVs), which is adjacent to the striatum (Obernier and Alvarez-Buylla, 2019). These two regions are considered neurogenic zones of the adult brain, as they have been shown to comprise NS/PCs that are capable of generating distinct cell types *in vitro* as well as *in vivo* (Lie et al., 2005; Bizen et al., 2014). However, adult NS/PCs proliferate at a much slower pace than during embryogenesis (Furutachi et al., 2013). In addition to the two neurogenic regions of the adult CNS, there is good evidence that during ischemia, multiple neurogenic sites within the brain parenchyma are activated, lasting for more than 1 month after the induction of ischemic injury (Kokaia et al., 2006).

Neurogenesis, together with gliogenesis, largely depends on molecular and genetic inputs such as growth factors and cellular signaling pathways, creating a microenvironment, or niche, for NS/PCs. Moreover, these processes are also modulated during pathological states (O’Keeffe et al., 2009; Bowman et al., 2013; Lamus et al., 2020). The role of the canonical Wnt signaling pathway in the brain development has been well established (Chenn and Walsh, 2002; Machon et al., 2003, 2007; Kalani et al., 2008; Borday et al., 2018; Chodelkova et al., 2018). Nevertheless, recent research has directed its attention more on the function of this pathway in postnatal neurogenesis, and in the modulation of the properties of adult NS/PCs (Lie et al., 2005). The Wnt ligands belong to a group of secreted cysteine-rich glycosylated proteins that are involved in cellular processes, such as cell proliferation and differentiation, synaptic plasticity, or programmed cell death (Wiese et al., 2018; Palomer et al., 2019). In the absence of a Wnt signal, the multi-protein destruction complex is formed in the cytoplasm. Kinases of this complex put a molecular tag on β-catenin, the key factor of the whole cascade, and thus mark it for degradation in the proteasome. On the other hand, activation of the pathway stabilizes β-catenin. This stabilization is achieved via a negative regulation of glycogen synthase kinase 3β (GSK-3β), followed by the accumulation of β-catenin in the cytoplasm and its subsequent translocation to the nucleus, where it binds to the transcription factors T-TCF/LEF, and thus influences expression of Wnt target genes (Nusse and Clevers, 2017). Many of these genes are implicated in the proliferation and differentiation of neural precursors, or in self-regulation of the pathway, with its numerous negative feedback loops (Mikels and Nusse, 2006; ten Berge et al., 2008).

Wnt signaling influences the fate of postnatal NS/PCs. The activation of β-catenin-dependent transcription promotes the proliferation of precursor cells in the SVZ, while its inhibition reduces the number of newly generated cells (Adachi et al., 2007). Moreover, Wnt7a ligand increases the count of cells expressing neuronal markers, while suppressing gliogenesis (Prajerova et al., 2010). Similar effects of active Wnt signaling were also observed in our previous study on neonatal NS/PCs (Kriska et al., 2016). Additionally, it has been shown that hypoxia and FCI increase the number of NS/PCs in the hippocampus and SVZ, promote the expansion of neuroblasts 1 month after MCAO, and that canonical Wnt signaling is involved in this process (Cui et al., 2011; Zhang et al., 2014; Knotek et al., 2020). The importance of Wnt signaling in the differentiation of NS/PCs to neurons under ischemic conditions was also documented by Zhang et al. (2016). Congruent with these findings, Zhang and Chopp (2016) highlighted the stroke-induced activation of NS/PCs, and their subsequent differentiation to neuroblasts, in their review. Moreover, Shruster et al. (2012) disclosed that Wnt signaling contributed to functional recovery following FCI in mice.

The aim of this study was to assess the effect of the canonical Wnt signaling pathway on the differentiation potential of NS/PCs under physiological conditions and following ischemia. Our data show that FCI increases the expression of Wnt target genes and cell-type-specific proteins, and concomitantly influences the electrophysiological properties of differentiated NS/PCs. Moreover, we found that the effect of active Wnt signaling on the differentiation of NS/PCs isolated from adult mice is greater after the induction of FCI, and that it promotes neurogenesis at the expense of gliogenesis. Therefore, we suggest that modulating Wnt signaling might ameliorate the negative effects associated with ischemic brain injury.

## Materials and Methods

### Transgenic Animal Models

**FIGURE 1:**
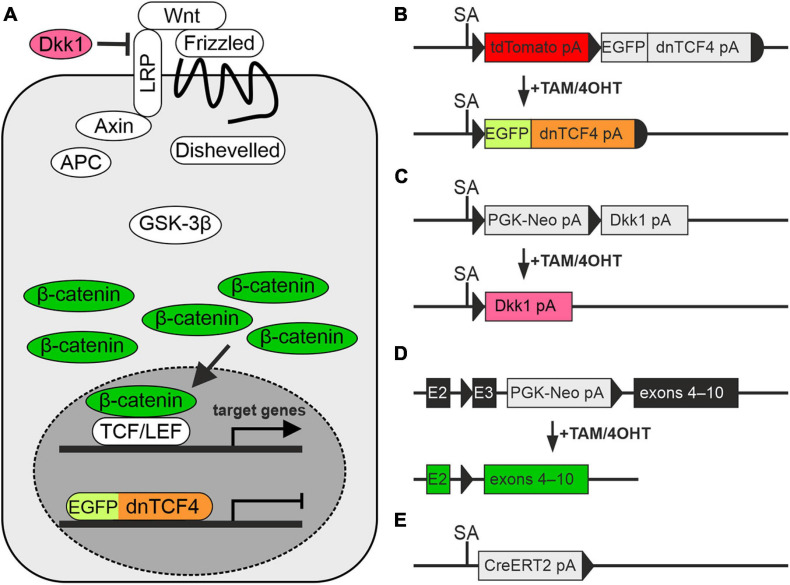
Modulation of the Wnt signaling pathway in transgenic mice. **(A)** A simplified scheme of the canonical Wnt signaling pathway that shows by color-coding where the alterations in the cascade take place. **(B–E)** Schemes depicting genetic modifications present in the mouse strains used in the study. **(B)** The transcription of the Wnt-responsive genes in the nucleus is blocked by dominant negative T-Cell Factor 4 (dnTCF4; orange) fused to the enhanced green fluorescent protein (EGFP; light green). **(C)** Suppression of Wnt signaling at the membrane receptor level, due to the overexpression of a secreted Wnt inhibitor Dickkopf 1 (Dkk1; dark pink). **(D)** Activation of the pathway by the production of a stable variant of β-catenin. Cre-mediated excision of exon 3 (E3) removes the amino acid sequence involved in the degradation of the protein, and thus stable β-catenin aberrantly hyper-activates Wnt target genes (dark green). **(E)** Cre deletor mouse *Rosa26-CreERT2* carries the gene encoding tamoxifen/4-hydroxytamoxifen (TAM/4OHT)-inducible Cre recombinase, fused with a mutated form of the estrogen receptor (CreERT2). APC, adenomatous polyposis coli; E2-3, exons 2-3; GSK-3β, glycogen synthase kinase 3β; LRP, low-density lipoprotein receptor-related protein; pA, polyadenylation site; PGK-Neo, neomycin resistance cassette; SA, splice acceptor site; TCF/LEF, T-Cell Factor/Lymphoid Enhancer Factor; td, tandem dimer; Wnt, Wingless/Integrated.

In our experiments, we used transgenic mouse strains that served as a source of NS/PCs, and as a tool for manipulating the canonical Wnt signaling pathway. The procedures involving the use of laboratory animals were carried out in accordance with the European Communities Council Directive from November 24, 1986 (86/609/EEC) and with the guidelines of the Institute of Experimental Medicine, Czech Academy of Sciences, which was approved by the Animal Care Committee (approval numbers 18/2011, 146/2013, and 2/2017). All efforts were made to minimize both the suffering and the numbers of mice assigned to the individual experiments. Young adult (postnatal day 50–56) males of three different transgenic mouse strains were utilized. These animals facilitated the manipulation of the canonical Wnt signaling pathway at different subcellular levels, specifically at the nuclear, membrane and cytoplasmic level (Figure 1A). First, we used the *Rosa26-tdTomato-EGFP/dnTCF4* mice (Janeckova et al., 2016) that produced dominant negative (dn)TCF4 protein from the *Rosa26* locus upon a Cre-mediated excision of a transcriptional blocker that was located upstream of *dnTCF4* cDNA. The dnTCF4 protein acted as a Wnt signaling inhibitor in the cell nucleus (Figure 1B). Moreover, this strain was designed to produce tandem dimeric (td) red fluorescent protein Tomato that was replaced by EGFP after Cre-mediated DNA excision. The *Rosa26-Dkk1* mice (Wu et al., 2008) were then used. After the Cre-mediated excision of a transcriptional blocker, the extracellular Wnt pathway inhibitor Dickkopf 1 (Dkk1) was produced from the ubiquitous *Rosa26* locus (Figure 1C), sequestering membrane receptors and thus inhibiting the pathway. Lastly, the *Catnb^*lox(ex*3)^* mice harbored the floxed allele of the *Ctnnb1* gene encoding protein β-catenin (Harada et al., 1999). In these mice the allele enabled conditional stabilization of β-catenin (Figure 1D). All these mouse strains were individually crossbred with the general Cre deletor mouse *Rosa26-CreERT2* that possessed tamoxifen (TAM)-inducible Cre recombinase fused with a ERT2 (Ventura et al., 2007; Figure 1E). According to the resulting genotype, the mice enabled the inhibition of Wnt signaling, either at the nuclear (genotype *Rosa26^*dnTCF*4/CreERT2^*; further termed ‘dnTCF4’ mice/cells) or membrane level (genotype *Rosa26^*Dkk*1/CreERT2^*; further termed ‘Dkk1’ mice/cells). Conversely, the *Ctnnb1^*del(ex*3)/^*^+^*Rosa26*^+^*^/CreERT2^* mice (further termed ‘Ex3’ mice/cells) produced a stable form of β-catenin protein that hyper-activated the canonical Wnt signaling pathway upon the Cre-mediated excision of the DNA sequence encoding exon 3 of the *Ctnnb1* gene. The genetic background of all mouse strains was C57BL/6. We used wild-type (WT) C57BL/6 mice for single-cell RNA sequencing (scRNA-seq).

The Cre-recombination-mediated manipulation of Wnt signaling was achieved either *in vitro*, by the addition of 1 μM (Z)-4-hydroxytamoxifen (4OHT; Sigma-Aldrich, St. Louis, MO, United States) dissolved in ethanol (EtOH) into the differentiation medium or *in vivo*, by intraperitoneal (i.p.) injections of TAM (200 mg/kg of the animal’s body weight; Toronto Research Chemicals, Toronto, ON, Canada) dissolved in corn oil (CO; Sigma-Aldrich, St. Louis, MO, United States). Tamoxifen injections were delivered in two doses, each once a day for two consecutive days, and experiments (induction of FCI or tissue collection) were performed on the third day after the last TAM injection. Cell cultures/mice treated only with the vehicle (EtOH or CO) were considered controls and labeled ‘EtOH’ and ‘CO,’ respectively, while cell cultures/mice with manipulated Wnt signaling were labeled ‘4OHT’ or ‘TAM.’

### The Induction of Focal Cerebral Ischemia

Mice undergoing permanent MCAO, a procedure that has become a conventional model of FCI (Colak et al., 2011), were anesthetized with 2% isoflurane (Abbot, IL, United States) and maintained at 1% isoflurane using a vaporizer (Tec-3, Cyprane Ltd., Keighley, United Kingdom). An incision in the skin between the orbit and the external auditory meatus was made and the temporal muscle was retracted. A ∼1.5 mm hole was then drilled through the frontal bone, ∼1.0 mm rostrally to the fusion of the zygoma and the squamosal bone, and ∼3.5 mm ventrally to the dorsal surface of the brain. After the *dura mater* was opened and removed, the middle cerebral artery was exposed and cauterized with a pair of bipolar tweezers (SMT, Prague, Czechia) at a proximal location. The body temperature of the mouse was maintained at 37 ± 1°C using a heating pad throughout the surgery. After the operation, the mice were injected with 0.5 ml of saline solution subcutaneously. Analgesics were administered when necessary. The operated mice were labeled ‘MCAO,’ while the non-operated animals were considered controls and labeled ‘CTRL’. Previously, we analyzed coronal brain slices using tetrazolium chloride staining and detected no ischemic lesions in either the non-operated mice or sham-operated animals, which were subjected to the same surgery procedure as the MCAO mice but without dura opening and vessel occlusion (Honsa et al., 2014). This distal model of MCAO has a high survival rate (>95%) and good reproducibility, as it typically yields an infarct lesion of a relatively small volume only in the cortical region (Honsa et al., 2013). To facilitate the orientation in the text, we provide a summarizing table of all combinations of the treatments used in our experiments in Table 1.

**TABLE 1:** Treatments applied on neural stem/progenitor cells.

**Experiment**	**Ischemia**	**Wnt signaling**
*In vitro*	CTRL	EtOH
		4OHT
	MCAO	EtOH
		4OHT
*In vivo*	CTRL	CO
		TAM
	MCAO	CO
		TAM

**Altogether eight different combinations of treatments were used in our analyses, depending on the type of an experiment, the (patho)physiological conditions of the brain, and the state of the canonical Wnt signaling pathway. 4OHT, (Z)-4-hydroxytamoxifen; CO, corn oil; CTRL, control; EtOH, ethanol; MCAO, middle cerebral artery occlusion; TAM, tamoxifen*.*

### Tissue Isolation and Cell Culture Preparation

To prepare tissue or cell culture specimens for subsequent analyses, we prepared coronal sections of mouse brains and cut out lateral SVZs for further processing. Primary cultures were derived from NS/PCs isolated from the SVZs of the LVs; from both the right and left hemispheres from CTRL mice, while only from the SVZ ipsilateral to the site of injury in mice 3 days after MCAO (Supplementary Figure 1).

The animals were deeply anesthetized first with 4% isoflurane and subsequently with pentobarbital solution (PTB; 100 mg/kg, i.p.; Sigma-Aldrich, St. Louis, MO, United States), and perfused transcardially with an ice-cold isolation buffer containing (in mM): 110 *N*-Methyl-D-glucamine (NMDG)-Cl, 2.5 KCl, 24.5 NaHCO_3_, 1.25 Na_2_HPO_4_, 0.5 CaCl_2_, 7 MgCl_2_, 20 glucose, osmolality 290 ± 3 mOsmol/kg. After subsequent decapitation, the brains were quickly removed from the skull and sliced into ∼500 μm coronal slices using vibratomes HM 650 V (MICROM International GmbH, Walldorf, Germany) or Leica VT 1200S (Baria, Prague, Czechia) and the SVZs were carefully dissected out and cut into smaller pieces using a razor. To obtain a single-cell suspension, the tissue was first incubated with continuous shaking at 37°C for 45 min in 1 ml of papain solution (20 U/ml; Worthington, Lakewood, NJ, United States) with 0.2 ml of deoxyribonuclease (DNase; Worthington, Lakewood, NJ, United States). After papain treatment, the activity of the enzyme was inhibited with 1 ml of ovomucoid inhibitor solution (Worthington, Lakewood, NJ, United States). The tissue was then mechanically dissociated by gentle trituration using a 1 ml pipette and centrifuged at 1,020 × *g* for 3 min. After centrifugation, the supernatant was discarded and the cells were resuspended in 1 ml of proliferation medium containing Neurobasal-A medium (Life Technologies, Waltham, MA, United States), supplemented with the B27 supplement (B27; 2%; Life Technologies, Waltham, MA, United States), L-glutamine (2 mM; Sigma-Aldrich, St. Louis, MO, United States), antimicrobial reagent primocin (100 μg/ml; InvivoGen, Toulouse, France), and bFGF (10 ng/ml), and EGF (30 ng/ml); both were purchased from PeproTech, Rocky Hill, NJ, United States. The cells were subsequently filtered through a 70 μm cell strainer, into a 100 mm-diameter Petri dish containing an additional 9 ml of proliferation medium. The cells were cultured as neurospheres, at 37°C and 5% CO_2_. After ∼12 days of *in vitro* proliferation, the formed neurospheres were collected and transferred into a 12 ml Falcon tube, and centrifuged at 1,020 × *g* for 3 min. The supernatant was discarded, and 1 ml of protease trypsin (Sigma-Aldrich, St. Louis, MO, United StatesA) was added to the pelleted neurospheres. After 3 min of trypsin incubation, 1 ml of trypsin inhibitor (Sigma-Aldrich, St. Louis, MO, United States) was added to the dissociated cells to block the proteolytic effect of trypsin. Subsequently, a negligible portion (∼100 μl) of the cell suspension was used to count cells in the hemocytometer. The rest of the suspension was centrifuged at 1,020 × *g* for 3 min and plated on poly-L-lysine (PLL)-coated (Sigma-Aldrich, St. Louis, MO, United States) coverslips placed in a 24-well plastic plate at the cell density of 6 × 10^4^ cells/cm^2^ in a differentiation medium. The differentiation medium had the same composition as the proliferation medium, but it was devoid of EGF and with a twofold (20 ng/ml) concentration of bFGF. The cells were cultured at 37°C and 5% CO_2_, with a medium exchange on every third day. After 8–9 days of *in vitro* differentiation, the cells were used for electrophysiological measurements and immunocytochemical staining. To estimate the impact of Wnt signaling inhibition or activation on the differentiation of NS/PCs, 4OHT-treated cultures were compared with the corresponding EtOH-treated cells.

In order to prepare tissue slices for immunohistochemical analyses, the mice were deeply anesthetized with PTB (100 mg/kg, i.p.) and perfused transcardially using a saline solution (ArdeaPharma, a.s., Sevetin, Czechia) with 0.65% heparin (Zentiva Group, Prague, Czechia) at room temperature (RT), and subsequently with ice-cold 4% paraformaldehyde (PFA; Sigma-Aldrich, St. Louis, MO, United States). The brain was then left in PFA for 3 more hours for thorough fixation, with subsequent transfer to the sucrose gradient for cryoprotection. The tissue was incubated in 10% sucrose for 12 h, then in 20% sucrose for 24 h, followed by 30% sucrose incubation for a further 72 h. Coronal sections of 30 μm were prepared using a cryostat (Leica CM1850, Leica Microsystems, Wetzlar, Germany) and subsequently stored at −20°C in a cryopreservation solution composed of 30% glycerol and 30% ethylene glycol in a PBS.

Tissue for RT-qPCR was isolated as described in Supplementary Figure 1. Briefly, the mice were anesthetized with 4% isoflurane and subsequently with 1% PTB and perfused transcardially with an ice-cold isolation buffer. After decapitation, the brains were quickly removed from the skull and cut into ∼500 μm coronal sections using a vibrating microtome. The SVZs were dissected out and cut into smaller pieces using a razor. Finally, the tissue was transferred into 2 ml Eppendorf tubes containing TRI Reagent (Sigma-Aldrich, St. Louis, MO, United States) for further RT-qPCR analysis.

To prepare a single-cell suspension for FACS and subsequent scRNA-seq, the tissue was isolated according to Supplementary Figure 1. The WT C57BL/6 mice were anesthetized with 1% PTB and perfused transcardially with an ice-cold isolation buffer containing (in mM): 136.0 NaCl, 5.4 KCl, 10.0 4-(2-hydroxyethyl)-1-piperazineethanesulfonic acid (HEPES), 5.5 glucose, with pH 7.4 and osmolality 290 ± 3 mOsmol/kg. After decapitation, the brains were quickly removed from the skull and cut into ∼750 μm coronal sections using a vibrating microtome. The SVZs with the adjacent striatum were dissected out and cut into smaller pieces using a razor. To obtain a single-cell suspension, the tissue was first incubated with continuous shaking at 37°C for 40 min in 1 ml of papain dissolved in the isolation buffer (20 U/ml) supplemented with 0.3 ml of DNase. After the papain treatment, the tissue was mechanically dissociated by gentle trituration using a 1 ml pipette and centrifuged at 300 × *g* for 3 min. After centrifugation, the supernatant was discarded and the cells were resuspended in 0.9 ml of the isolation buffer supplemented with 100 μl of an ovomucoid inhibitor solution and 50 μl of DNase. Afterward, the cells were centrifuged at 300 × *g* for 3 min, the supernatant was discarded, and the cell pellet was resuspended in Dulbecco’s PBS (Sigma-Aldrich, St. Louis, MO, United States) and filtered through a 70 μm pre-separation filter (Miltenyi Biotec, Bergisch Gladbach, Germany). The single-cell suspension was then processed using a debris removal solution (Miltenyi Biotec, Bergisch Gladbach, Germany) according to the manufacturer’s protocol for smaller amounts of tissue. The resulting cell suspension was resuspended in Neurobasal-A medium and kept at 4°C for further processing.

### Electrophysiological Recordings

The electrophysiological properties of *in vitro* differentiated cells were recorded using the patch-clamp technique in the whole-cell configuration. Recording micropipettes with a tip resistance of ∼8–10 MΩ were made from borosilicate glass capillaries (Sutter Instruments, Novato, CA, United States) using a P-97 Brown-Flaming puller (Sutter Instruments, Novato, CA, United States) and subsequently filled with an artificial intracellular solution containing (in mM): 10 HEPES, 130 KCl, 0.5 CaCl_2_, 2 MgCl_2_, 5 ethylene glycol-bis(β-aminoethyl ether)-N,N,N′,N′-tetraacetic acid (EGTA), with pH 7.2, and in some cases also mixed with Alexa Fluor hydrazide 488 (A488; Molecular Probes, Carlsbad, CA, United States) for the visualization of recorded cells. The measurements were made in an aCSF containing (in mM): 122 NaCl, 3 KCl, 1.5 CaCl_2_, 1.3 MgCl_2_, 1.25 Na_2_HPO_4_, 28 NaHCO_3_, and 10 D-glucose (osmolality 300 ± 5 mmol/kg) and this solution was continuously gassed with 5% CO_2_ to maintain a final pH of 7.4. All the recordings were made at RT on coverslips perfused with aCSF in the recording chamber of an upright Axioscop microscope (Zeiss, Gottingen, Germany), equipped with 2 electronic micromanipulators (Luigs & Neumann, Ratingen, Germany), and a high-resolution AxioCam HRc digital camera (Zeiss, Gottingen, Germany). Electrophysiological data were measured with a 10 kHz sample frequency using EPC9 or EPC10 amplifiers controlled by PatchMaster software (HEKA Elektronik, Lambrecht/Pfalz, Germany) and filtered using a Bessel filter.

The values of the *V*_*M*_ were measured by switching the EPC9/10 amplifiers to the current-clamp mode. Using the FitMaster software (HEKA Elektronik, Lambrecht/Pfalz, Germany), IR was calculated from the current value at 40 ms after the onset of the depolarizing 10 mV pulse from the holding potential of −70 mV. *C*_*M*_ was determined automatically from the Lock-in protocol by the software. Current patterns were obtained by hyperpolarizing and depolarizing the cell membrane from the holding potential of −70 mV to the values ranging from −160 mV to 40 mV in 10 mV steps, while the duration of each pulse was 50 ms. The inwardly rectifying potassium (*K*^+^; *K*_*IR*_), the *K*_*A*_, and the *K*_*DR*_ current components were determined as follows. In order to isolate the *K*_*DR*_ current components, a voltage step from −70 to −60 mV was used to subtract the time- and voltage-independent currents. To activate the *K*_*DR*_ currents only, the cells were held at −50 mV, and the amplitude of the *K*_*DR*_ currents was measured at 40 mV, 40 ms after the onset of the pulse. The *K*_*IR*_ currents were determined analogously at −140 mV, also 40 ms after the onset of the pulse, while the cells were held at −70 mV. The *K*_*A*_ currents were measured at 40 mV and were isolated by subtracting the current traces, clamped at −110 mV from those clamped at −50 mV, and its amplitude was measured at the peak value. While measuring TTX-sensitive sodium (Na^+^) currents, the cells were held at −70 mV, and the current amplitudes were isolated by subtracting the current traces measured in a solution containing 1 μM TTX (Alomone Labs, Jerusalem, Israel) from those measured in the absence of TTX in aCSF. The Na^+^ current amplitudes were measured at the peak value. The current densities were calculated by dividing the maximum current amplitudes by the corresponding *C*_*M*_ values for each individual cell. The action potentials were obtained in the current-clamp mode and current values ranged from 50 to 1,000 pA in 50 pA steps, and the pulse duration was 300 ms.

After recording, the cells on the coverslips were fixed in 4% PFA dissolved in 0.2 M PB (pH 7.4) for 9 min, then transferred to 10 mM PBS (pH 7.2) and stored at 4°C for post-recording identification using immunofluorescence staining.

### Immunochemistry and Confocal Microscopy

The coverslips with cells or 30-μm-thick coronal brain slices were washed 3 times for 10 min in PBS. They were then rinsed for 2 h at 4°C in blocking solution containing 5% Chemiblocker (Millipore, Billerica, MA, United States) and 0.5% Triton X-100 (Sigma-Aldrich, St. Louis, MO, United States) diluted in 10 mM PBS. The same blocking solution was used as the diluent for all primary and secondary antibodies. The specimens were incubated overnight at 4°C with primary antibodies. The following primary antibodies were used: rabbit polyclonal anti-GFAP (1:800; Sigma-Aldrich, St. Louis, MO, United States), mouse monoclonal anti-GFAP (1:800; Sigma-Aldrich, St. Louis, MO, United States) conjugated to cyanine dye 3 (Cy3), rabbit polyclonal anti-PDGFRα (1:200; Santa Cruz Biotechnology, Dallas, TX, United States), rabbit polyclonal anti-DCX (1:1000; Abcam, Cambridge, United Kingdom), mouse monoclonal anti-MAP2 (1:800; Merck Millipore, Billerica, MA, United States), mouse monoclonal anti-PCNA (1:1000; Abcam, Cambridge, United Kingdom). The next day, the specimens were washed 3 times for 10 min with PBS, which was followed by incubation with secondary antibodies for a further 2 h at 4°C. The secondary antibodies were goat polyclonal anti-rabbit/mouse IgG conjugated to A488, or Alexa Fluor 594 or 660 (A594/660; 1:200; Molecular Probes, Carlsbad, CA, United States). Next, the specimens were washed 3 times for 10 min in PBS and rinsed for 5 more minutes, at RT, in 300 nM 4′,6-diamidino-2-phenylindole (DAPI; Molecular Probes, Carlsbad, CA, United States) diluted in PBS for cell nuclei visualization. At the end of the procedure, the specimens were mounted onto microscope slides using Aqua Poly/Mount (Polysciences Inc., Eppelheim, Germany). Once the mounting medium solidified, the specimens were ready to be analyzed by confocal microscopy.

An LSM 5 DUO confocal fluorescence microscope (Zeiss, Gottingen, Germany), equipped with an Arg/HeNe laser and a 40× oil objective, was used for immunofluorescence analyses and, furthermore, the fluorescence signals were analyzed using the ImageJ software (NIH, Bethesda, MD, United States). Superimposed images of fluorescent stainings were obtained by overlaying several individual confocal planes. The images were subsequently digitally filtered and the immunopositive areas were used for quantification. The areas corresponding to the immunoreactivity of the cells in *in vitro* cultures were calculated in random regions of interest, and divided by the DAPI-positive area to normalize them to the cell number. Cells for immunocytochemical staining against β-catenin were fixed for 10 min in 4% PFA, permeabilized with 0.25% Triton X-100 for another 10 min, and washed in PBS. Overnight incubation with primary mouse monoclonal antibody (1:2000; BD Transduction Laboratories, San Jose, CA, United States) at 4°C was followed by incubation with goat anti-mouse secondary antibody conjugated to A488 dye for 1 h at RT. Finally, DAPI (Sigma-Aldrich, St. Louis, MO, United States) was used for counterstaining. Fluorescent microscopy images were taken from EtOH and 4OHT cultures. The A488 and DAPI fluorescence signals were analyzed with the ImageJ software, and the intensity of β-catenin staining was normalized to the cell number in every image. The obtained data were evaluated by Student’s *t*-test.

The differentiation potential of progenitor cells of the SVZ *in situ* in coronal sections, was assessed by utilizing a spinning disk confocal fluorescent microscope Dragonfly 530 Andor (Oxford Instruments, Oxford, United Kingdom), which was equipped with Zyla 4.2 PLUS sCMOS camera and Fusion acquisition system. Superimposed images of GFAP, DCX, and PCNA stainings were obtained by overlaying 6–12 individual confocal images captured by a 20× objective. A series of the images was then digitally fused using the Fusion stitching tool. The obtained images were processed in the Imaris visualization software (Oxford Instruments, Oxford, United Kingdom). The immunopositive SVZ areas were dissected as shown in Supplementary Figure 2 and quantified using Fiji ImageJ software (NIH, Bethesda, MD, United States). The areas corresponding to immunopositive cells of the SVZ were calculated and normalized to the DAPI-positive area using a macro written in the IJM scripting language for the Fiji software package (fiji.sc). First, the confocal data were split into individual channels. Next, segmentation of DAPI-stained nuclei was done by thresholding dialog and joined segmented nuclei were split by a watershed procedure. The number of the nuclei was then counted by the Particle Analyzer in Fiji. A similar segmentation approach of thresholding was also applied to the antibody channels. Finally, we identified and counted individual antibody-positive cells using the Image Calculator in Fiji that can create a picture with intersects of two images, and the Particle Analyzer that is capable of counting the numbers of the overlaps in the whole SVZ. Altogether six SVZ sections from two biological replicates were analyzed in each immunostaining data set.

### Reverse Transcription Quantitative Polymerase Chain Reaction

From tissue samples, RNA was purified using a TRI Reagent (Sigma-Aldrich, St. Louis, MO, United States) or RNA Blue (Top-Bio, Prague, Czechia) according to the manufacturers’ protocol. The reverse transcription of RNA and RT-qPCR were performed using LightCycler 480 SYBR Green I Master (Roche Diagnostics, Indianapolis, IN, United States). The primers for RT-qPCR are listed in Supplementary Table 1.

### Single-Cell RNA Sequencing and Fluorescence-Activated Cell Sorting

The single-cell suspension for the scRNA-seq analysis was prepared as described above. Prior to sorting, the single-cell suspension derived from the WT C57BL/6 mice was stained for 15 min for lymphocytes and endothelial cells (CD45 and CD31; BioLegend Way, San Diego, CA, United States), and living cells (CellTrace calcein green; Thermo Fisher Scientific, Waltham, MA, United States). All antibodies were diluted in Neurobasal-A medium supplemented with 2% B27 and after staining, cells were washed in Neurobasal-A medium and kept at 4°C until sorting. Viable (Hoechst 33258-negative (Life Technologies, Carlsbad, CA, United States) and CellTrace-calcein green-positive) and CD45/CD31-negative cells were sorted using FACS (BD Influx, San Jose, CA, United States). The single cells were sorted into 1.5 ml Eppendorf tubes containing 200 μl of Neurobasal-A medium supplemented with 2% B27, and analyzed using a scRNA-seq approach.

The Chromium System (10× Genomics, Pleasanton, CA, United States) was used to generate barcoded single-cell cDNA libraries. The barcoded cDNA was then pooled and sequenced using the NextSeq 500 high-throughput sequencing system (Illumina, San Diego, CA, United States). The sequencing data were analyzed using a strategy for comprehensive integration of single-cell data developed by Stuart et al. (2019), and the identified cell clusters were matched to the molecular atlas of cells obtained from the adult SVZ published by Mizrak et al. (2019). Genes with significantly increased expression in the corresponding cluster, when compared to all other cells in the sample, are listed in Supplementary Table 2.

### Data Analysis

The data are presented as means ± S.E.M. or as means ± standard deviation (S.D.) for a number (n) of specimens/cells. The Student’s *t*-test was used to determine significant differences between the two experimental groups, and one-way or two-way ANOVA with Tukey’s *post hoc* test, was performed to determine significant differences among more experimental groups. The significance was calculated in the GraphPad Prism software (San Diego, CA, United States), and the values of *p* < 0.05 were considered significant (^∗^, one asterisk), *p* < 0.01 very significant (^∗∗^, two asterisks), and *p* < 0.001 extremely significant (^∗∗∗^, three or more asterisks).

## Results

This study is a logical continuation of our previous experiments performed on neonatal mice under physiological conditions, where we revealed that canonical Wnt signaling promotes neurogenesis at the expense of gliogenesis (Kriska et al., 2016). In order to gain a deeper insight into the differentiation of adult NS/PCs, we assessed here the impact of the Wnt signaling pathway under physiological conditions, as well as following the induction of FCI in the adult mouse brain. To elucidate the effect of the Wnt/β-catenin pathway on the differentiation potential of NS/PCs in non-operated (CTRL) mice and mice after the induction of permanent FCI (MCAO), we compared either *in vitro* cultures treated with 4OHT to their respective controls (EtOH), or tissue specimens from mice with manipulated Wnt signaling (TAM), to control mice with intact Wnt signaling (CO). We used a set of experimental approaches to disclose the effect of Wnt signaling manipulation on the mRNA, protein, and functional levels.

### Cell Types Originated From Adult Neural Stem/Progenitor Cells Differentiated *in vitro*

**FIGURE 2:**
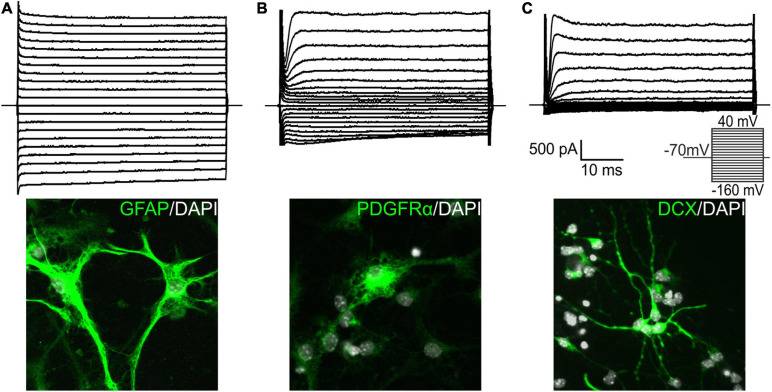
Characterization of cell types identified among differentiated neural stem/progenitor cells. We identified three distinct cell types in the *in vitro* cultures, and supplemented them with illustrative images. Cells with a passive current pattern **(A)** were considered astrocytes as they were mostly GFAP-positive. They displayed predominantly time- and voltage-independent *K*^+^ currents, together with small amplitudes of delayed outwardly rectifying *K*^+^ currents (*K*_*DR*_) and inwardly rectifying *K*^+^ currents (*K*_*IR*_). Cells displaying a complex current profile and expressing mainly PDGFRα **(B)** were considered precursor cells. They expressed fast activating and inactivating outwardly rectifying *K*^+^ currents (*K*_*A*_), *K*_*DR*_, and *K*_*IR*_ currents. Doublecortin (DCX)-positive cells with an outwardly rectifying current pattern **(C)** were considered neuron-like cells, or neuroblasts, and expressed *K*_*A*_ and *K*_*DR*_ currents. Current patterns were obtained by hyper- and depolarizing the cell membrane from the holding potential of –70 mV to the values ranging from –160 to 40 mV in 10 mV increments (see the inset). DAPI, 4′,6-diamidino-2-phenylindole; GFAP, glial fibrillary acidic protein; PDGFRα, platelet-derived growth factor receptor alpha.

Based on electrophysiological properties, together with previous post-recording immunocytochemical identification (Kriska et al., 2016), of EtOH-treated CTRL cells, we identified three cell types that represented *in vitro* differentiated adult NS/PCs. GFAP-positive astrocytes with a passive current pattern representing time- and voltage-independent *K*^+^ currents possessed a relatively high average *V*_*M*_ (−85.26 ± 0.43 mV; *n* = 148) and low IR (73.45 ± 3.01 MΩ; *n* = 148; Figure 2A). PDGFRα-positive precursor cells with a complex current pattern expressed *K*_*IR*_ currents, together with *K*_*DR*_ and *K*_*A*_ currents (Figure 2B). They had *V*_*M*_ similar to astrocytes (−86.87 ± 0.58 mV; *n* = 127) and IR of medium values (150.92 ± 7.42 MΩ; *n* = 127). DCX- or MAP2-positive neuron-like cells (or neuroblasts) with an outwardly rectifying current profile composed of *K*_*A*_ currents and *K*_*DR*_ currents (Figure 2C) were characterized by *V*_*M*_ of −86.26 ± 0.86 mV (*n* = 174) and the highest IR values (1056.29 ± 66.21 MΩ; *n* = 174).

### Analysis of the Effects of Wnt Pathway Modulation in Adult NS/PCs Differentiated *in vitro*

**FIGURE 3:**
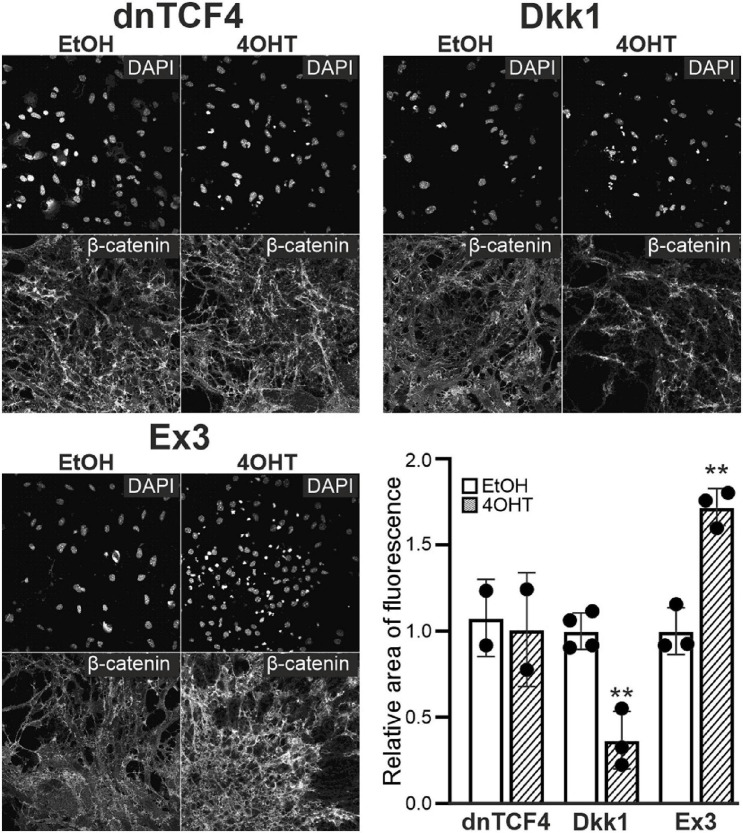
Changes of β-catenin protein level upon Wnt pathway modulation. Representative fluorescence images show DAPI and β-catenin staining in differentiated adult neural stem/progenitor cells with inhibited (dnTCF4 and Dkk1), or activated (Ex3) Wnt signaling. Cells were only treated with either ethanol (EtOH) or with (Z)-4-hydroxytamoxifen (4OHT) dissolved in EtOH, and analyzed 8 days after the onset of *in vitro* differentiation. Diagram indicates quantification of β-catenin expression, showing the proportion of the area of positively-stained cells to the DAPI-positive area (*n* = 12). The values are represented as mean ± S.D. (standard deviation). Statistical significance was calculated using *t*-test; **p < 0.01. DAPI, 4′,6-diamidino-2-phenylindole; Dkk1, Dickkopf 1; dnTCF4, dominant negative T-cell factor 4; Ex3, exon 3; n, number.

Next, we analyzed NS/PCs differentiated *in vitro* utilizing immunocytochemical staining against β-catenin, the principal element of the Wnt signaling; the NS/PCs were derived from “our” mouse models that enabled the Wnt signaling pathway manipulation. We did not detect any changes in the expression of this protein after Wnt signaling inhibition by dnTCF4 expression, as the manipulation of Wnt signaling took place in the nucleus; nevertheless, we identified its decreased amounts after Wnt signaling inhibition at the membrane receptor level. As expected, in cells with hyper-activated Wnt signaling, the expression of the protein was increased (Figure 3).

**FIGURE 4:**
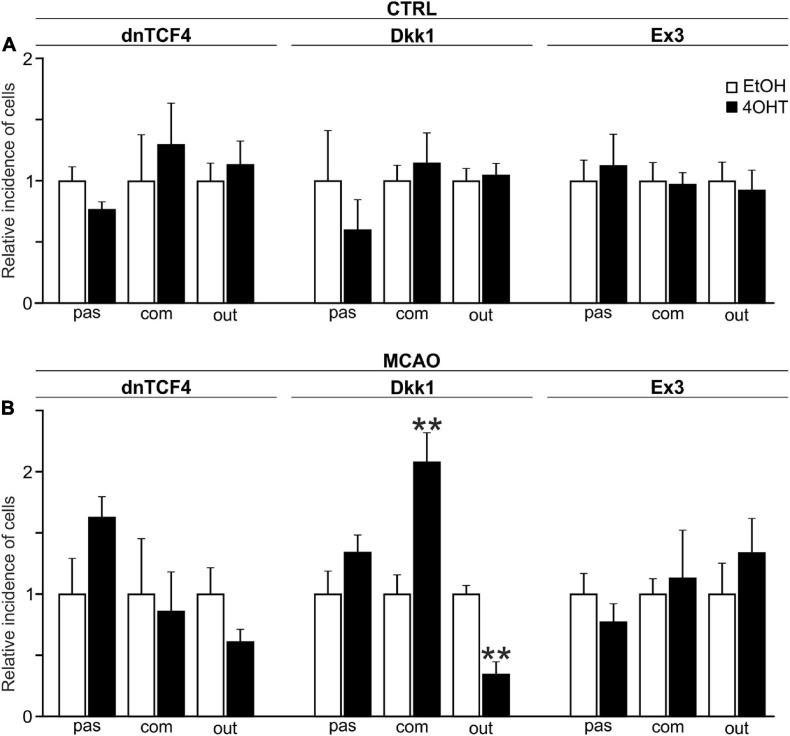
Changes in the incidence of GFAP-, PDGFRα-, and DCX-positive cells after Wnt signaling manipulation in intact and ischemic mice. The incidence of cells showing passive (pas; GFAP^+^ cells), complex (com; PDGFRα^+^ cells) or outwardly rectifying (out; DCX^+^ cells) current patterns was examined in neural stem/progenitor cell (NS/PC) cultures derived from adult control, non-operated (CTRL; **A**) as well as operated (MCAO; **B**) mice. The relative incidence of cells in controls was arbitrarily set to 1. Sixteen founder mice were used to derive NS/PCs from each mouse strain. The incidence was quantified from the following total number of cells (in brackets): CTRL-dnTCF4-EtOH (145), CTRL-dnTCF4-4OHT (141), CTRL-Dkk1-EtOH (116), CTRL-Dkk1-4OHT (114), CTRL-Ex3-EtOH (152), CTRL-Ex3-4OHT (197), MCAO-dnTCF4-EtOH (75), MCAO-dnTCF4-4OHT (72), MCAO-Dkk1-EtOH (75), MCAO-Dkk1-4OHT (79), MCAO-Ex3-EtOH (80), and MCAO-Ex3-4OHT (80). The incidence of cells in controls (EtOH) was compared to the incidence of the same cell types in cells with manipulated Wnt signaling (4OHT) with Student’s *t*-test. **p < 0.01. 4OHT, (Z)-4-hydroxytamoxifen; DCX, doublecortin; Dkk1, Dickkopf 1; dnTCF4, dominant negative T-cell factor 4; EtOH, ethanol; Ex3, exon 3; GFAP, glial fibrillary acidic protein; MCAO, middle cerebral artery occlusion; PDGFRα, platelet-derived growth factor receptor alpha.

The incidence of the three cell types identified by the electrophysiological experiments was assessed by the patch-clamp technique in 413 EtOH- and 452 4OHT-treated cells from CTRL mice. Having analyzed the data from CTRL cells, we failed to identify any alterations in the cell incidence caused by the Wnt signaling pathway manipulation (Figure 4A). Nevertheless, 3 days after MCAO we found significant changes in the cultures with inhibited Wnt signaling at the membrane receptor level (Figure 4B). In these cultures, we observed decreased numbers of cells showing an outwardly rectifying current pattern [from 49.17 ± 3.61% in EtOH (*n* = 37) to 16.91 ± 5.11% in 4OHT (*n* = 13)] and an increased count of cells with a complex current profile [from 20.42 ± 3.25% in EtOH (*n* = 15) to 42.35 ± 4.93% in 4OHT (*n* = 34)].

The electrophysiological characterization of membrane properties was conducted utilizing the patch-clamp technique measurements on *in vitro* differentiated NS/PCs. Altogether 1,355 cells were analyzed, and the effect of Wnt signaling manipulation in CTRL and MCAO mice was investigated in cells with a passive, complex and outwardly rectifying current profile (Tables 2–7).

**TABLE 2:** Membrane properties of differentiated adult NS/PCs isolated from CTRL mice, and showing a passive current profile.

	**dnTCF4**		**Dkk1**		**Ex3**	
	**EtOH**	**4OHT**	**EtOH**	**4OHT**	**EtOH**	**4OHT**
***V*_*M*_ [mV]**	−84.50.7	−83.01.0	−86.80.9	−85.51.0	**−85.4 ± 0.7**	−87.6 ± 0.6*
**IR [M**Ω** ]**	67.64.2	66.72.8	**49.1 ± 3.1**	75.3 ± 4.5***	89.45.0	79.24.0
***C*_*M*_ [pF]**	27.12.3	30.42.9	**31.4 ± 2.8**	21.7 ± 1.7**	22.61.8	20.71.4
***K*_*IR*_/*C*_*M*_ [pA/pF]**	2.90.3	2.50.4	2.40.7	3.20.4	3.50.9	2.70.3
***K*_*DR*_/*C*_*M*_ [pA/pF]**	4.00.8	6.31.5	7.42.3	8.73.0	5.81.0	3.90.6
***n***	62	55	25	25	61	63

4OHT, (Z)-4-hydroxytamoxifen; C_*M*_, membrane capacitance; CTRL, control, non-operated mice; Dkk1, Dickkopf 1; dnTCF4, dominant negative T-cell factor 4; EtOH, ethanol; Ex3, exon 3; IR, input resistance; K_*DR*_, delayed outwardly rectifying K^+^ currents; K_*IR*_, inwardly rectifying K^+^ currents; K_*IR*_/C_*M*_, K_*DR*_/C_*M*_, current densities; n, number of cells; NS/PCs, neural stem/progenitor cells; V_*M*_, membrane potential. Values in bold indicate significant differences between EtOH- and 4OHT-treated cultures; *p < 0.05, **p < 0.01, ***p < 0.001.

**TABLE 3:** Membrane properties of differentiated adult NS/PCs isolated from MCAO mice, and showing a passive current profile.

	**dnTCF4**		**Dkk1**		**Ex3**	
	**EtOH**	**4OHT**	**EtOH**	**4OHT**	**EtOH**	**4OHT**
***V*_*M*_ [mV]**	**−84.9 ± 0.7**	−87.1 ± 0.4**	**−86.0 ± 0.6**	−89.1 ± 0.3***	−86.10.3	−85.50.6
**IR [M**Ω** ]**	50.13.6	47.21.9	44.43.5	46.32.5	45.92.1	48.32.8
***C*_*M*_ [pF]**	40.65.0	36.32.4	46.64.7	36.53.5	53.59.5	51.410.6
***K*_*IR*_/*C*_*M*_ [pA/pF]**	2.40.4	1.90.3	2.90.6	4.20.6	2.00.3	2.10.3
***K*_*DR*_/*C*_*M*_ [pA/pF]**	5.91.1	4.20.5	5.61.0	4.60.7	5.80.7	4.70.7
***n***	25	40	23	32	39	30

4OHT, (Z)-4-hydroxytamoxifen; C_*M*_, membrane capacitance; Dkk1, Dickkopf 1; dnTCF4, dominant negative T-cell factor 4; EtOH, ethanol; Ex3, exon 3; IR, input resistance; K_*DR*_, delayed outwardly rectifying K^+^ currents; K_*IR*_, inwardly rectifying K^+^ currents; K_*IR*_/C_*M*_, K_*DR*_/C_*M*_, current densities; MCAO, middle cerebral artery occlusion; n, number of cells; NS/PCs, neural stem/progenitor cells; V_*M*_, membrane potential. Values in bold indicate significant differences between EtOH- and 4OHT-treated cultures; **p < 0.01, ***p < 0.001.

**TABLE 4:** Membrane properties of differentiated adult NS/PCs isolated from CTRL mice, and showing a complex current profile.

	**dnTCF4**		**Dkk1**		**Ex3**	
	**EtOH**	**4OHT**	**EtOH**	**4OHT**	**EtOH**	**4OHT**
***V*_*M*_ [mV]**	**−82.9 ± 1.0**	−88.6 ± 0.7***	−89.00.9	−86.90.9	**−88.8 ± 0.8**	−86.5 ± 0.7*
**IR [M**Ω** ]**	138.212.8	153.013.8	136.49.7	136.39.0	**175.6 ± 14.0**	138.6 ± 7.7*
***C*_*M*_ [pF]**	**16.9 ± 1.6**	12.7 ± 0.8*	14.51.1	15.21.3	15.71.2	16.61.1
***K*_*IR*_/*C*_*M*_ [pA/pF]**	6.10.6	7.70.9	8.30.8	10.71.2	**7.9 ± 0.7**	4.5 ± 0.5***
***K*_*DR*_/*C*_*M*_ [pA/pF]**	34.95.1	52.89.0	54.16.3	76.09.6	**42.1 ± 5.5**	25.6 ± 2.9**
***K*_*A*_/*C*_*M*_ [pA/pF]**	22.34.3	20.83.7	**45.4 ± 6.3**	25.1 ± 2.7*	19.24.3	11.42.4
**Na^+^/*C*_*M*_ [pA/pF]**	28.212.4	39.55.4	11.85.1	52.90.0	19.73.5	27.35.7
***n***	43	45	39	38	45	58

4OHT, (Z)-4-hydroxytamoxifen; C_*M*_, membrane capacitance; CTRL, control, non-operated mice; Dkk1, Dickkopf 1; dnTCF4, dominant negative T-cell factor 4; EtOH, ethanol; Ex3, exon 3; IR, input resistance; K_*A*_, fast activating and inactivating outwardly rectifying K^+^ currents; K_*DR*_, delayed outwardly rectifying K^+^ currents; K_*IR*_, inwardly rectifying K^+^ currents; K_*IR*_/C_*M*_, K_*DR*_/C_*M*_, K_*A*_/C_*M*_, Na^+^/C_*M*_, current densities; n, number of cells; NS/PCs, neural stem/progenitor cells; V_*M*_, membrane potential. Values in bold indicate significant differences between EtOH- and 4OHT-treated cultures; **p < 0.01, ***p < 0.001.

**TABLE 5:** Membrane properties of differentiated adult NS/PCs isolated from MCAO mice, and showing a complex current profile.

	**dnTCF4**		**Dkk1**		**Ex3**	
	**EtOH**	**4OHT**	**EtOH**	**4OHT**	**EtOH**	**4OHT**
***V*_*M*_ [mV]**	**−84.7 ± 1.3**	−88.2 ± 0.7*	**−87.2 ± 0.7**	−89.4 ± 0.6*	−86.00.5	−85.21.3
**IR [M**Ω** ]**	171.933.5	120.716.3	115.426.3	111.810.6	93.111.4	110.611.9
***C*_*M*_ [pF]**	21.43.5	20.33.3	**23.3 ± 2.4**	15.2 ± 1.2**	34.513.8	18.92.9
***K*_*IR*_/*C*_*M*_ [pA/pF]**	6.11.0	6.31.2	**6.3 ± 1.4**	11.7 ± 1.6*	**5.1 ± 0.9**	9.0 ± 1.4*
***K*_*DR*_/*C*_*M*_ [pA/pF]**	61.39.4	52.410.7	32.78.0	79.616.7	59.415.1	54.012.0
***K*_*A*_/*C*_*M*_ [pA/pF]**	15.94.9	27.28.7	26.59.5	39.07.9	38.713.5	26.311.7
**Na^+^/*C*_*M*_ [pA/pF]**	−−	−−	−−	−−	−−	−−
***n***	15	13	15	34	23	23

4OHT, (Z)-4-hydroxytamoxifen; C_*M*_, membrane capacitance; Dkk1, Dickkopf 1; dnTCF4, dominant negative T-cell factor 4; EtOH, ethanol; Ex3, exon 3; IR, input resistance; K_*A*_, fast activating and inactivating outwardly rectifying K^+^ currents; K_*DR*_, delayed outwardly rectifying K^+^ currents; K_*IR*_, inwardly rectifying K^+^ currents; K_*IR*_/C_*M*_, K_*DR*_/C_*M*_, K_*A*_/C_*M*_, Na^+^/C_*M*_, current densities; MCAO, middle cerebral artery occlusion; n, number of cells; NS/PCs, neural stem/progenitor cells; V_*M*_, membrane potential. Values in bold indicate significant differences between EtOH- and 4OHT-treated cultures; *p < 0.05, **p < 0.01.

**TABLE 6:** Membrane properties of differentiated adult NS/PCs isolated from CTRL mice, and showing an outwardly rectifying current profile.

	**dnTCF4**		**Dkk1**		**Ex3**	
	**EtOH**	**4OHT**	**EtOH**	**4OHT**	**EtOH**	**4OHT**
***V*_*M*_ [mV]**	**−85.5 ± 1.6**	−79.4 ± 1.8*	**−86.9 ± 1.3**	−81.8 ± 1.2**	**−86.2 ± 1.5**	−77.8 ± 1.8***
**IR [M**Ω** ]**	997.197.3	887.596.0	**959.7 ± 97.2**	352.4 ± 30.2***	**1217.6 ± 134.3**	877.8 ± 91.9*
***C*_*M*_ [pF]**	8.40.8	9.50.6	**9.1 ± 0.5**	16.1 ± 1.2***	**9.1 ± 0.6**	11.1 ± 0.8*
***K*_*DR*_/*C*_*M*_ [pA/pF]**	112.27.1	118.39.0	139.68.9	150.514.1	101.24.7	88.55.8
***K*_*A*_/*C*_*M*_ [pA/pF]**	84.57.6	75.512.3	**110.9 ± 9.8**	40.1 ± 7.2***	**80.6 ± 7.3**	44.0 ± 7.1***
**Na^+^/*C*_*M*_ [pA/pF]**	−−	24.212.8	**9.8 ± 1.1**	19.1 ± 4.3*	12.42.6	14.44.3
***n***	47	47	65	57	62	61

4OHT, (Z)-4-hydroxytamoxifen; C_*M*_, membrane capacitance; CTRL, control, non-operated mice; Dkk1, Dickkopf 1; dnTCF4, dominant negative T-cell factor 4; EtOH, ethanol; Ex3, exon 3; IR, input resistance; K_*A*_, fast activating and inactivating outwardly rectifying K^+^ currents; K_*DR*_, delayed outwardly rectifying K^+^ currents; K_*DR*_/C_*M*_, K_*A*_/C_*M*_, Na^+^/C_*M*_, current densities; n, number of cells; NS/PCs, neural stem/progenitor cells; V_*M*_, membrane potential. Values in bold indicate significant differences between EtOH- and 4OHT-treated cultures; *p < 0.05, **p < 0.01, ***p < 0.001.

**TABLE 7:** Membrane properties of differentiated adult NS/PCs isolated from MCAO mice, and showing an outwardly rectifying current profile.

	**dnTCF4**		**Dkk1**		**Ex3**	
	**EtOH**	**4OHT**	**EtOH**	**4OHT**	**EtOH**	**4OHT**
***V*_*M*_ [mV]**	**−77.4 ± 2.4**	−85.7 ± 1.4*	−78.12.3	−84.32.1	**−80.1 ± 2.5**	−67.0 ± 3.4**
**IR [M**Ω** ]**	1112.2132.8	1028.2123.1	1166.2157.2	741.8132.1	1230.8228.2	1018.7156.6
***C*_*M*_ [pF]**	8.90.4	8.80.3	9.00.4	7.91.3	10.21.1	9.10.8
***K*_*DR*_/*C*_*M*_ [pA/pF]**	**100.4 ± 5.3**	149.3 ± 19.5**	109.56.7	137.117.1	99.36.6	114.610.9
***K*_*A*_/*C*_*M*_ [pA/pF]**	80.86.2	95.512.1	**103.7 ± 10.1**	180.3 ± 30.1**	87.512.3	94.919.4
**Na^+^/*C*_*M*_ [pA/pF]**	11.60.3	15.72.3	12.35.5	−	−	31.910.4
***n***	34	19	37	13	18	24

4OHT, (Z)-4-hydroxytamoxifen; C_*M*_, membrane capacitance; Dkk1, Dickkopf 1; dnTCF4, dominant negative T-cell factor 4; EtOH, ethanol; Ex3, exon 3; IR, input resistance; K_*A*_, fast activating and inactivating outwardly rectifying K^+^ currents; K_*DR*_, delayed outwardly rectifying K^+^ currents; K_*DR*_/C_*M*_, K_*A*_/C_*M*_, Na^+^/C_*M*_, current densities; MCAO, middle cerebral artery occlusion; n, number of cells; NS/PCs, neural stem/progenitor cells; V_*M*_, membrane potential. Values in bold indicate significant differences between EtOH- and 4OHT-treated cultures; *p < 0.05, **p < 0.01.

In cells with a passive current pattern, the changes in the membrane properties were sporadic (Tables 2, 3). Wnt signaling manipulation only affected the passive electrophysiological properties. The strongest effect was identified in the *V*_*M*_ following the induction of FCI, where we discovered that following Wnt signaling inhibition, the cells became hyperpolarized. In dnTCF4 mice, the average *V*_*M*_ changed from −84.9 ± 0.7 mV (*n* = 25) to −87.1 ± 0.4 mV (*n* = 40), while in Dkk1 mice, its value changed from −86.0 ± 0.6 mV (*n* = 23) to −89.1 ± 0.3 mV (*n* = 32). Such hyperpolarization was observed in all analyzed cell types that were isolated from ischemic tissue. In CTRL mice, we recorded higher IR after Wnt signaling inhibition at the membrane receptor level [from 49.1 ± 3.1 MΩ (*n* = 25) to 75.3 ± 4.5 MΩ (*n* = 25)], together with lowered *C*_*M*_ [from 31.4 ± 2.8 pF (*n* = 25) to 21.7 ± 1.7 pF (*n* = 25)]. Although we did not observe any differences in the current densities of *K*^+^ currents, we detected a decreased incidence of cells showing *K*_*IR*_ currents following MCAO (data not shown). These results might correspond to astrocyte differentiation and their proliferative capacity following FCI.

In cells with a complex current pattern, we observed opposite effects of Wnt signaling inhibition and activation on the values of *V*_*M*_ (Tables 4, 5). Inhibition of the pathway resulted in hyperpolarization [from −82.9 ± 1.0 mV (*n* = 43) to −88.6 ± 0.7 mV (*n* = 45) in CTRL dnTCF4 mice, and from −84.7 ± 1.3 mV (*n* = 15) to −88.2 ± 0.7 mV (*n* = 13) and from −87.2 ± 0.7 (*n* = 15) to −89.4 ± 0.6 mV (*n* = 34) in MCAO dnTCF4 and Dkk1 mice, respectively], while Wnt signaling activation led to depolarization of the membrane [from −88.8 ± 0.8 mV (*n* = 45) to −86.5 ± 0.7 mV (*n* = 58) in CTRL Ex3 mice]. Besides the passive membrane properties, Wnt signaling manipulation also influenced the expression of voltage-gated *K*^+^ channels in this cell type. After Wnt signaling activation in CTRL mice, the densities of all examined *K*^+^ currents decreased [significant decrease in *K*_*IR*_, from 7.9 ± 0.7 pA/pF (*n* = 44) to 4.5 ± 0.5 pA/pF (*n* = 57), and in *K*_*DR*_, from 42.1 ± 5.5 pA/pF (*n* = 44) to 25.6 ± 2.9 pA/pF (*n* = 58)]. This effect of Wnt signaling subsided after the induction of FCI, which is in accordance with our observation that under ischemic conditions, the majority of *K*^+^ currents were upregulated, independently on the Wnt signaling inhibition/activation [significant increase in *K*_*IR*_ from 6.3 ± 1.4 pA/pF (*n* = 15) to 11.7 ± 1.6 pA/pF (*n* = 34) in Dkk1 mice and from 5.1 ± 0.9 pA/pF (*n* = 23) to 9.0 ± 1.4 pA/pF (*n* = 22) in Ex3 mice].

In cells displaying an outwardly rectifying current pattern, the effect of Wnt signaling inhibition on the *V*_*M*_ was reversed in CTRL and MCAO mice (Tables 6, 7). In CTRL mice, the membrane was depolarized [from −85.5 ± 1.6 mV (*n* = 47) to −79.4 ± 1.8 mV (*n* = 47) in dnTCF4 mice and from −86.9 ± 1.3 mV (*n* = 65) to −81.8 ± 1.2 mV (*n* = 57) in Dkk1 mice]. Conversely, after the induction of FCI, the cells became hyperpolarized [significantly in dnTCF4 mice, from −77.4 ± 2.4 mV (*n* = 34) to −85.7 ± 1.4 mV (*n* = 19)], which was observed in all three cell types. The opposite impact of the Wnt signaling pathway on the *V*_*M*_ coincides with the expression of *K*^+^ channels mediating outward currents. After Wnt signaling inhibition, the current densities of *K*_*DR*_ and *K*_*A*_ were decreased or increased only negligibly in CTRL mice [significant decrease in *K*_*A*_, from 110.9 ± 9.8 pA/pF (*n* = 51) to 40.1 ± 7.2 pA/pF (*n* = 33), in Dkk1 mice] while in MCAO mice, their current densities increased [significant increase in *K*_*DR*_ in dnTCF4 mice, from 100.4 ± 5.3 pA/pF (*n* = 34) to 149.3 ± 19.5 pA/pF (*n* = 19), and significant increase in *K*_*A*_ in Dkk1 mice, from 103.7 ± 10.1 pA/pF (*n* = 35) to 180.3 ± 30.1 pA/pF (*n* = 10)]. A higher efflux of *K*^+^ ions out of the cell could potentially explain the hyperpolarization observed after the induction of FCI in cells with an outwardly rectifying as well as a complex current profile.

Additionally, we identified changes in the incidence of neuron-like cells expressing voltage-dependent Na^+^ channels (data not shown). Their counts increased both after Wnt signaling activation (from 3 to 11 cells; total numbers in both CTRL and MCAO cultures) and its inhibition in the cell nucleus (from 3 to 9 cells). Alternatively, the attenuation of the Wnt pathway at the membrane receptor level resulted in a smaller number of these cells (from 10 to 7 cells). Interestingly, the same trends were found in *in vitro* cultures derived from both CTRL as well as MCAO mice. Finally, the density of Na^+^ currents was only significantly changed in non-operated Dkk1 mice. We identified its increase [from 9.8 ± 1.1 pA/pF (*n* = 8) in EtOH to 19.1 ± 4.3 pA/pF (*n* = 7) in 4OHT] in cells with an outwardly rectifying current pattern, which could add to the depolarization we observed in this cell type.

The electrophysiological analysis gave us an outline of the effect of Wnt signaling on the functional properties of differentiated NS/PCs. Wnt manipulation had a minimal impact on the electrophysiological properties of cells with a passive current pattern. Conversely, the most significant changes were identified after FCI in cells with a complex and an outwardly rectifying current profile. We particularly observed hyperpolarized cells after Wnt signaling inhibition, which can be explained by higher current densities of *K*_*DR*_ and *K*_*A*_. Furthermore, we only detected a few cells with voltage-dependent Na^+^ channels. These results imply that Wnt signaling affects the distribution of *K*^+^ and Na^+^ channels, and thus influences the membrane properties of differentiated cells.

By comparing the current densities in control (CTRL-TAM) and post-ischemic (MCAO-TAM) animals, in which the Wnt signaling pathway was modified, we found that the blockage of Wnt signaling at the nuclear level (dnTCF4) had no effect on the current densities in any of the three cell types (Tables 2–7). On the other hand, Wnt signaling inhibition at the membrane receptor level (Dkk1) significantly increased the current densities of *K*_*A*_ channels in cells with a complex current pattern (from 25.1 ± 2.7 pA/pF to 39.0 ± 7.9 pA/pF; Tables 4, 5) as well as in those displaying an outwardly rectifying current profile (from 40.1 ± 7.2 pA/pF to 180.3 ± 30.1 pA/pF; Tables 6, 7). We hypothesize that the diverse effect of Wnt signaling inhibition at different subcellular levels can be caused by the compensatory effect of endogenous Tcf4 produced in dnTCF4 mice (Janeckova et al., 2016). Interestingly, following FCI, Wnt signaling hyper-activation (Ex3) increased current densities of *K*_*IR*_ (from 4.5 ± 0.5 pA/pF to 9.0 ± 1.4 pA/pF), *K*_*DR*_ (from 25.6 ± 2.9 pA/pF to 54.0 ± 12.0 pA/pF), and *K*_*A*_ (from 11.4 ± 2.4 pA/pF to 26.3 ± 11.7 pA/pF) in cells with a complex current pattern (Tables 4, 5), and *K*_*DR*_ (from 88.5 ± 5.8 pA/pF to 114.6 ± 10.9 pA/pF), *K*_*A*_ (from 44.0 ± 7.1 pA/pF to 94.9 ± 19.4 pA/pF) and Na^+^ (from 14.4 ± 4.3 pA/pF to 31.9 ± 10.4 pA/pF) current densities in cells showing an outwardly rectifying current pattern (Tables 6, 7). In cells with a passive current profile (Tables 2, 3), Wnt signaling modulation following FCI had no impact on the current densities of voltage-dependent *K*^+^ channels. This could be explained by the fact that the expression of voltage-dependent *K*^+^ channels in cells showing a passive current pattern (Tables 2, 3) is relatively lower than their expression in other cell types (Tables 4–7). Additionally, there was an IR decline in post-ischemic passive cells, presumably suggesting a reduced expression of two-pore domain *K*^+^ channels (Zhou et al., 2009); however, this decline occurred regardless of Wnt signaling inhibition/activation (Tables 2, 3).

### Analysis of the Effects of Wnt Pathway Modulation in the Adult Brain

Changes in β-catenin protein levels upon Wnt signaling manipulation were assessed by immunoblotting of brain tissue lysates prepared from SVZs of adult mice of corresponding genotypes. However, the densitometric analysis revealed no consistent changes in the expression of total β-catenin or its non-phosphorylated, active form (data not shown). Additionally, we analyzed the expression of putative Wnt target genes, namely *Axin2*, naked cuticle homolog 1 (*Nkd1*), and *Troy* [alternative name tumor necrosis factor receptor superfamily, member 19 (*Tnfrsf19*)]. Overall, their expression was either downregulated or not significantly changed after Wnt signaling manipulation in CTRL mice of all strains (Supplementary Figure 3). The expression pattern was similar in MCAO mice; however, a significantly increased expression of the most Wnt target genes was observed. Moreover, hyper-activation of Wnt/β-catenin signaling induced by β-catenin stabilization was evident only in case of the *Axin2* gene (Supplementary Figure 3). These results indicate that brain tissue used for the analyses is more heterogeneous than cell cultures as it contains, in addition to NS/PCs (and their descendants), also other cell types that might react to Wnt signaling manipulation differently.

**FIGURE 5:**
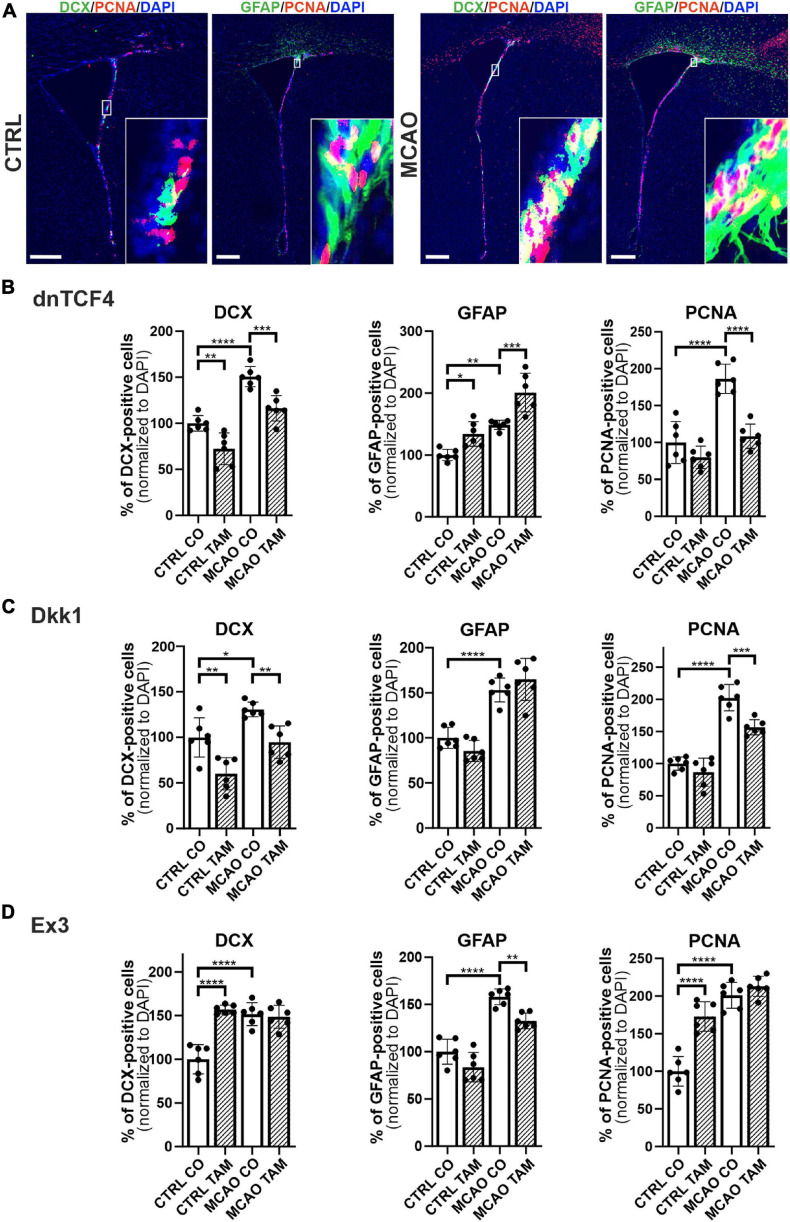
Immunohistochemical analysis of changes in the production of cell-type-specific markers in the brain of transgenic mice after FCI. **(A)** Representative images of the SVZ isolated from CTRL and MCAO mice without Wnt pathway modulation. Increased stainings of neuroblasts marked by doublecortin (DCX), glial fibrillary acidic protein (GFAP)-positive astrocytes, and dividing cells harboring proliferating cell nuclear antigen (PCNA) were recorded 3 days after the induction of ischemia. Scale bar = 0.2 mm. Modulation of the canonical Wnt signaling pathway changed the incidence of cell types under control, physiological conditions (CTRL) as well as 3 days upon focal cerebral ischemia induced by middle cerebral artery occlusion (MCAO). Diagrams show quantification of immunohistochemical staining in the SVZ of CTRL and MCAO mice only treated with either tamoxifen (TAM) or vehicle, corn oil (CO). Tamoxifen-induced production of dominant negative human T-cell factor protein (dnTCF4; **B**) and Dickkopf 1 Wnt inhibitory protein (Dkk1; **C**) were used to achieve Wnt signaling inhibition and, conversely, production of constitutively active β-catenin (Ex3; **D**) was initiated in order to obtain Wnt pathway activation. Experiments were performed using two biological replicates and three technical replicates for each treatment (*n* = 6). Average values of the control mice (CTRL CO) were arbitrary set to 100% of immunogenic signal. Error bars represent standard deviation and one-way ANOVA was used to determine significant differences among the experimental groups; *p < 0.05, **p < 0.01, ***p < 0.001, ****p < 0.0001. Ex3, exon 3.

Therefore, we performed immunohistochemical analysis in coronal brain sections where we analyzed the expression of DCX, GFAP, and a marker of dividing cells, PCNA, in the SVZ. In the vehicle-treated mice, we observed higher overall immunopositivity for all marker proteins after the induction of FCI (Figures 5A–D; due to the space constraints, the immunohistochemical staining is only shown for mice either non-operated or after FCI, indicated as ‘CTRL CO’ or ‘MCAO CO,’ respectively; for the immunohistochemical staining of all experimental groups, see Supplementary Figure 4). Furthermore, Wnt signaling inhibition caused higher expression of GFAP and lower expression of DCX in both CTRL and MCAO mice, while the immunopositivity of PCNA was decreased significantly only in MCAO mice (Figures 5B,C). These observations were more significant after Wnt signaling inhibition in the cell nucleus (Figure 5B). Conversely, Wnt signaling hyper-activation resulted in the overexpression of PCNA and DCX in CTRL mice, while this effect was diminished after FCI. Moreover, activation of the pathway led to the significantly decreased expression of GFAP only after the induction of FCI (Figure 5D).

**FIGURE 6:**
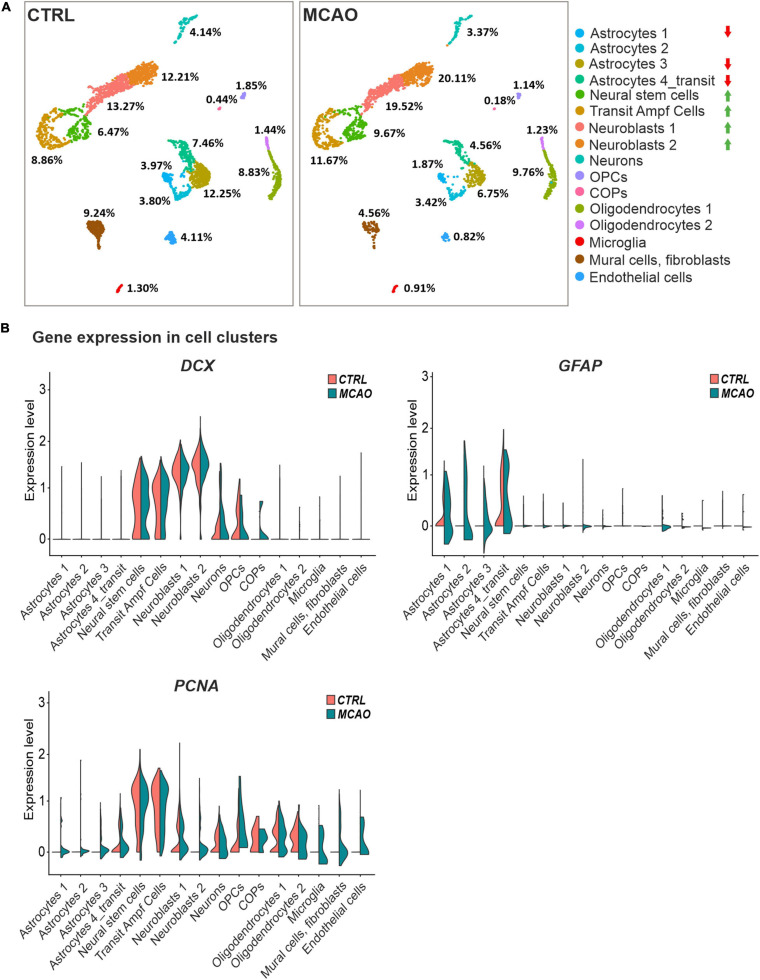
Single-cell RNA sequencing analysis of cells obtained from the SVZ and striatum of the healthy (CTRL) and ischemic brain (MCAO). **(A)** Cell distribution in individual clusters (subpopulations); clusters with the most significant decrease or increase in cell counts after focal cerebral ischemia (FCI) are indicated by red or green arrows, respectively. The percentage of cells present in different cell clusters is indicated; RNA sequencing was performed on 3,694 and 2,728 cells obtained from the control or ischemic brain, respectively. After removing apoptotic cells, the remaining 2,923 (CTRL) and 2,193 (MCAO) cells were analyzed. More than 124,206 reads were obtained from one cell on average, representing 2,420 genes in the CTRL sample. In the brain after MCAO, the numbers were 175,361 reads per cell, representing 2,646 genes. **(B)** “Violin” plots showing the expression of *DCX*, *GFAP*, and *PCNA* in the indicated cell clusters. COPs, committed oligodendrocyte precursors; CTRL, control; *DCX*, doublecortin; *GFAP*, glial fibrillary acidic protein; MCAO, middle cerebral artery occlusion; OPCs, oligodendrocyte precursor cells; *PCNA*, proliferating cell nuclear antigen.

To gain a deeper insight into the brain injury-induced cellular changes, we performed scRNA-seq of cells obtained from the brain of non-operated (CTRL) mice and animals 3 days after FCI. The SVZ (and adjacent striatum) was dissected, and the specimens were processed to obtain a single-cell suspension. The cells were subsequently FACS-sorted, and all living cells except leukocytes (CD45-positive cells) and vascular epithelium (CD31-positive) cells were used for the scRNA-seq analysis. The identified cell clusters were matched to the molecular atlas of cells obtained from the adult SVZ published by Mizrak et al. (2019) (Supplementary Table 2). Whereas the proportion of neural stem and transit amplifying cells, and two neuroblast subpopulations (neuroblasts 1 and 2), were after FCI increased, the number of matured neurons was reduced (Figure 6A). The latter observation could be attributed to the fact that the analyzed samples did not contain the site of injury in the cortex. Interestingly, the numbers of OPCs and COPs were reduced after FCI. Nevertheless, it should be noted that the OPCs and COPs counts were rather low in both situations. From four astrocyte subtypes, three of the subtypes (astrocytes 1 and 3, astrocytes 4_transit) were markedly decreased in brain tissue recovering from MCAO. Finally, a decrease in cells assigned to clusters representing mural cells (mainly pericytes) and fibroblasts was observed. After brain injury, pericytes contribute to the restoration of the blood-brain barrier. Immediately after brain damage, the number of pericytes decreases, but during the repair phase, pericytes undergo reactive pericytosis, i.e., the cells detach from cerebral blood vessels, change their shape and proliferate. Moreover, they form a cell mass demarcated by reactive astrocytes (Zehendner et al., 2015).

The *DCX* and *GFAP* transcripts were in CTRL mice and animals that underwent MCAO mainly distributed in the stem and progenitor cell clusters or in astrocytes, respectively. Additionally, *DCX* expression was emerging in COPs, supporting our previous observation that this subpopulation of neuron-glial antigen 2 (NG2)-positive glial cells might contribute to neurogenesis (Honsa et al., 2012). In contrast, FCI induced expression of proliferating cell marker *PCNA* in virtually all cell subpopulations (Figure 6B), which possibly relates to reactive gliosis after brain injury (Buffo et al., 2008).

**FIGURE 7:**
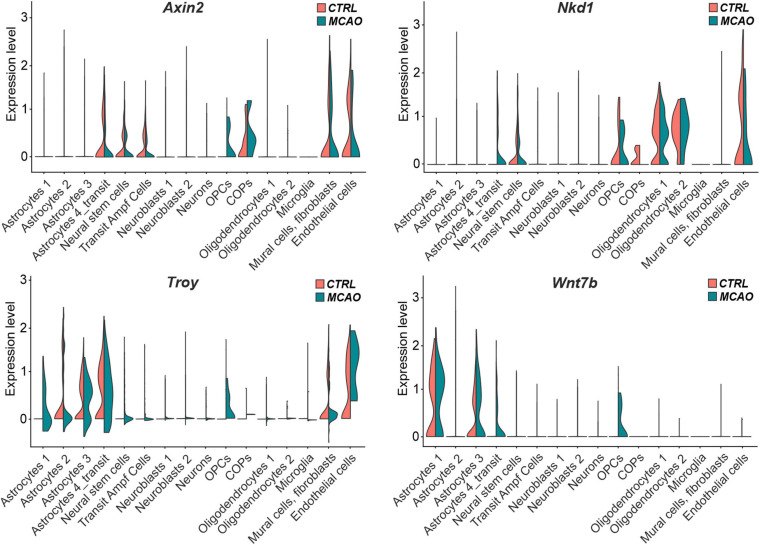
“Violin” plots showing expression of Wnt-responsive genes and *Wnt7b* mRNA in the indicated cell clusters. COPs, committed oligodendrocyte precursors; CTRL, control; MCAO, middle cerebral artery occlusion; *Nkd1*, naked cuticle homolog 1; OPCs, oligodendrocyte precursor cells; *Troy*, tumor necrosis factor receptor superfamily, member 19; *Wnt7b*, Wingless/Integrated 7b.

The analysis of the expression of the putative Wnt target genes *Axin2*, *Nkd1*, and *Troy* brought less consistent results (Figure 7). Whereas *Axin2* was in CTRL tissue expressed mainly in the transit subpopulation of astrocytes, neural stem cells, transit amplifying cells, and COPs; *Nkd1* was mainly produced in oligodendrocytes and their precursors (OPCs and COPs). In contrast, *Troy* expression was predominantly detected in three astrocyte (astrocytes 2–4) subpopulations. Additionally, *Axin2* and *Troy* mRNA was detected in brain mural cells and fibroblasts, while all the three genes were expressed in endothelial cells. In the brain tissue after FCI, we observed *de novo* expression of *Axin2* and *Nkd1* in OPCs and transit astrocytes, respectively. Moreover, *Troy* expression was upregulated in virtually all astrocyte subpopulations (Figure 7). Next, we analyzed the expression profiles of all 19 mammalian Wnt ligands in brain cells of CTRL and MCAO mice, and identified a relatively high expression of *Wnt7b*, a ligand which activates canonical Wnt signaling (Daneman et al., 2009; Posokhova et al., 2015). Interestingly, in the healthy brain, *Wnt7b* transcripts were limited to two astrocyte subpopulations (astrocyte 1 and 3); however, after brain injury, the ligand was induced in the transit astrocyte subtype (astrocyte 4_transit) and in OPCs (Figure 7, right bottom diagram).

## Discussion

### Transgenic Animal Models

Although the role of the canonical Wnt signaling pathway in neural development has been a main focus of countless studies, relatively little is known about its functions in adult neurogenesis and gliogenesis. This lack of information may stem from severe, constitutive dysregulations of this pathway that result in embryonic lethality (van Amerongen and Berns, 2006). To circumvent this issue, we used transgenic mouse strains that allowed TAM-induced, conditional manipulation of Wnt signaling. The same three mouse strains as in our previous study elucidating the effect of Wnt signaling on neonatal NS/PCs (Kriska et al., 2016), were used here; nevertheless, there were several differences in utilizing neonatal and adult mice in our experiments, as a variety of factors may influence the differentiation potential of adult NS/PCs. Among others, gender and age are the most important constituents in this process (Lembach et al., 2018; Vancamp et al., 2019). More specifically, clinical observations as well as experimental stroke confirmed that a high amount of estrogen in females acts as an endogenous neuroprotectant after the induction of cerebral ischemia (Hurn and Macrae, 2000), and that neurogenesis ceases with age (Lupo et al., 2019). For this reason, only male mice of a strictly defined age (postnatal day 50–56) were used in our experiments.

### The Effect of the Wnt Pathway Modulation in Adult NS/PCs Differentiated *in vitro*

The Cre/loxP system, together with the well-defined *in vitro* conditions, guaranteed highly reproducible means of either Wnt/β-catenin pathway hyper-activation (Ex3), or its inhibition at two different subcellular levels – in the cell nucleus (dnTCF4) or at the membrane receptor level (Dkk1; Figure 1). Additionally, we assessed the impact of Wnt signaling in CTRL as well as MCAO mice, where the differentiated NS/PCs were evaluated 3 days after the induction of FCI. This time point represents an earlier phase of ischemia, and the differentiation of precursor cells has a different outcome in later phases (Li et al., 2010).

Our primary cultures consisted of three cell types that were previously identified in experiments using neonatal mice (Kriska et al., 2016). These cell types comprised of GFAP-positive astrocytes, DCX/MAP2-positive neuron-like cells, and PDGFRα-positive precursor cells (Figure 2). The results from immunocytochemical staining against β-catenin in CTRL cell cultures (Figure 3) also resembled those obtained in neonatal animals, showing an elevated expression of the protein after Wnt signaling activation (Ex3), and only a decreased expression of β-catenin after inhibition by secreted Wnt signaling antagonist Dkk1. Additionally, the inhibition of Wnt signaling in the cell nucleus (dnTCF4) did not affect the levels of the β-catenin protein, which is in accordance with the fact that the alteration in the pathway occurs downstream of the β-catenin destruction complex. Nevertheless, the results from neonatal mice were more pronounced, which could be partially attributed to the transition from the neonatal to adult NS/PCs niche, with possible changes in the responsiveness to intrinsic or extrinsic stimuli (Morrison and Spradling, 2008; Urbán and Guillemot, 2014). The exact moment when the switch from embryonic to adult neurogenesis occurs is elusive, but it has been suggested that it is between the first and the third postnatal week in the rodent dentate gyrus (Pleasure et al., 2000).

Wnt signaling manipulation altered the passive membrane properties, as well as the expression of *K*^+^ channels in differentiated adult NS/PCs. We identified DCX/MAP2-positive neuron-like cells that showed the highest *K*_*A*_ and *K*_*DR*_ current densities (Tables 6, 7), which were comparable with previous studies from our laboratory (Prajerova et al., 2010). Moreover, we only characterized GFAP-positive astrocytes with very low densities of outwardly as well as inwardly rectifying *K*^+^ currents (Tables 2, 3). We observed decreased incidence of cells showing *K*_*IR*_ currents in NS/PCs-derived astrocytes following MCAO. Interestingly, a downregulation of *K*_*IR*_ channels was also identified in other types of neuropathologies (Bataveljić et al., 2012). *K*_*IR*_ expression is characteristic of mature astrocytes, and shifts these cells into the quiescent stages of the cell cycle (Higashimori and Sontheimer, 2007). These findings might signify that after the induction of FCI, astrocytes dedifferentiate and increase their proliferative activity. The other cell type represented PDGFRα-positive precursors that were defined by moderate values of *K*_*A*_, *K*_*DR*_, and *K*_*IR*_ (Tables 4, 5), with a sporadic expression of voltage-gated Na^+^ channels (data not shown). Similar membrane properties were also observed by Walz (2000). Additionally, we only identified significant changes in the density of Na^+^ currents of neuron-like cells in CTRL mice. The inability to detect any significant changes in the cultures derived from MCAO mice could be caused by the overall decreased expression of voltage-dependent Na^+^ channels after ischemia (Yao et al., 2002). However, another study identified exactly the opposite effect of ischemia on ion channels expression (Hernandez-Encarnacion et al., 2017). Regardless of the downregulation or upregulation, the effect of ischemic injury could overwhelm the effect of Wnt signaling manipulation in our model.

The incidence of the three cell types after Wnt signaling manipulation was not changed in the intact (CTRL) mice (Figure 4A). This could originate from the low responsiveness of these cells to Wnt signaling inhibition/activation, or in case of dnTCF4 and Dkk1 mice, by the relatively low level of the Wnt pathway inhibition caused by a compensatory effect of endogenous WT Tcf4 produced in dnTCF4 transgenic mice (Janeckova et al., 2016), or by the “dilution” of Dkk1 protein (Wu et al., 2008). Another reason for not detecting any changes in the cell incidence in CTRL cultures might also be the continuous, age-related depletion of the NS/PCs pool (Encinas et al., 2011) or the fact that NS/PCs prevail in a quiescent state (Codega et al., 2014). Thus, while Wnt inhibition adds to this BMP- and Notch-induced quiescence, the pathway activation in Ex3 mice might be ineffective in reverting the quiescent state (Urbán and Guillemot, 2014). Moreover, the majority of cells differentiated from NS/PCs undergo apoptosis and are rapidly phagocytized by microglia (Sierra et al., 2010). Interestingly, particular subpopulations of microglia have distinct effects on the differentiation potential of NS/PCs, as one subpopulation promotes astrogliogenesis and the other supports neurogenesis, while both inhibit NS/PCs proliferation (Vay et al., 2018). Nonetheless, the effect of Wnt signaling manipulation observed in neonatal cultures was (partially) reproduced with cells obtained from the adult brain after the induction of FCI. Specifically, Wnt signaling inhibition at the membrane receptor level suppressed neurogenesis (Figure 4B). The lower incidence of neuron-like cells corresponded with our observations from Western blotting experiments, where we identified a decrease in the expression of βIIItubulin, a neuronal marker, after Wnt signaling inhibition under ischemic conditions (data not shown). Why the changes in cell incidence were only observed in cell cultures originated from mice that underwent MCAO? The answer might be connected with the fact mentioned previously. Notch and BMP signaling maintain the quiescent (or dormant) state of adult NS/PCs. However, in response to brain ischemia, downregulation of Notch and BMP activity, and concomitant involvement of interferon gamma signaling, induce the activation/priming of NS/PCs (Llorens-Bobadilla et al., 2015). Another puzzling fact is the inconsistency between the effect of the two Wnt signaling inhibitors, dnTCF4 and Dkk1. An explanation could be the lower effectiveness of dnTCF4 in the Wnt pathway suppression and/or the contribution of the TCF/LEF-independent function(s) of β-catenin (Kelly et al., 2011; Valenta et al., 2012).

We obtained a relatively low yield of differentiated cells from adult mice, and this state did not improve even after a longer incubation of NS/PCs in proliferation medium. This phenomenon could be explained by the observation that adult NS/PCs are quiescent (Urbán and Guillemot, 2014). BMP signaling through the type IA receptor, which was identified in non-dividing cells of the adult hippocampus, is responsible for this behavior of precursor cells and active BMP signaling reversibly diminishes the proliferation of NS/PCs, while its inactivation only transiently enhances proliferation, with a subsequent reduction in the number of precursor cells (Mira et al., 2010). However, once young and old NS/PCs are activated, they exhibit similar proliferation and differentiation capacity (Kalamakis et al., 2019). Activated NS/PCs are characterized by the expression of EGFR (Codega et al., 2014). EGF is required for the increased proliferation of SVZ-derived cells (O’Keeffe et al., 2009). At the same time, NS/PCs might interconvert between the quiescent and activated state (Lugert et al., 2010; Codega et al., 2014). To enhance the cell yield, we increased the concentration of EGF in the proliferation medium by 50% (from 20 ng/ml in neonatal cultures to 30 ng/ml in adult cultures). However, to initiate the differentiation process of expanded NS/PCs, we had to omit this mitogenic agent in differentiation medium (Johe et al., 1996), which resulted in relatively low amounts of cells for electrophysiological measurements and other experiments.

### The Effect of Wnt Pathway Modulation in Adult NS/PCs Differentiated *in vivo*

Changes in the expression levels of the putative Wnt target genes in response to Wnt pathway modulation did not completely meet our expectations; particularly the upregulation of the gene expression after β-catenin stabilization in the Ex3 mice treated with TAM (Supplementary Figure 3). Since a mixture of cells isolated from the adult brain was used for the analysis, our original assumption was that the presence of other cell types “contaminating” the isolates did respond to Wnt signaling modulation differently than NS/PCs (Wallmen et al., 2012). However, subsequent analysis of expression profiles at the single-cell level provided a slightly more “prosaic” explanation. Like other evolutionarily conserved signaling pathways, the Wnt pathway does not always regulate the same group of genes and the transcriptional response to its activation (or inhibition) is thus tissue-specific; although there is a consensus that *Axin2* represents such a “universal” target gene (van de Moosdijk et al., 2020). The analysis of the expression levels of selected genes (*Axin2*, *Nkd1*, and *Troy*) in individual cell subpopulations clearly showed their different expression profile (Figure 7). Without experiments showing which target genes in the given brain region respond to Wnt pathway modulation, interpretation of the experiments described above will be complicated.

In order to disclose the differentiation potential of adult NS/PCs *in vivo*, we performed an exhaustive *in situ* immunohistochemical analysis of coronal slices with the SVZ region (Figure 5). This approach revealed an overall increased immunoreactivity of selected markers after FCI. Subsequent scRNA-seq showed that this increased positivity is mostly “due” to the cell subpopulations, which also produce the marker in healthy tissue (Figure 6B). Shruster et al. (2012) showed that stroke potently stimulates cell proliferation in the neurogenic niches, and that newly generated cells migrate to the injured striatum and cortex, while Wnt signaling promotes the survival of differentiated cells. Furthermore, we documented that Wnt signaling inhibition resulted in the abundance of GFAP in the SVZ, while, conversely, Wnt pathway hyper-activation leads to an overexpression of PCNA and DCX in the CTRL mice. This finding corresponds well with the data obtained from neonatal animals, where Wnt signaling activation, under physiological conditions, promoted neurogenesis at the expense of gliogenesis (Kriska et al., 2016). Likewise, the neurogenic effect of the Wnt pathway was also observed by Mastrodonato et al. (2018) and Kase et al. (2019), which corroborates our findings from the immunohistochemical analysis.

As already mentioned, the histological analysis of the SVZ showed an increase in GFAP-positive cells after brain injury. However, in the expression profiling at the individual cell level, we observed a decrease in cells in most subpopulations of GFAP-positive astrocytes. How to interpret this discrepancy? One explanation may be the fact that in order to obtain sufficient amounts of viable cells for the scRNA-seq analysis, cells were isolated not only from the SVZ but also from the adjacent striatum. Another possibility is that a subpopulation of GFAP-expressing cells represents proliferative astrocytes that have the potential to dedifferentiate and act as adult stem cells in the neurogenic regions of the adult brain (Seri et al., 2001). We might speculate that these cells still contain detectable amounts of GFAP protein; however, the corresponding transcripts are not detectable at the given depth of sequencing.

Finally, we observed no significant effect of Wnt signaling hyper-activation of the PCNA positivity in the brain after FCI. This fact might be explained by an overall increase in the cell proliferation induced by ischemia (Figure 6B). Importantly, scRNA-seq provided a lead for a further analysis of the role of Wnt signaling in the healthy and diseased brain. Wnt ligands are secreted cysteine-rich glycosylated proteins. The posttranslational modifications also include acylation performed by endoplasmic-reticulum-resident acyltransferase porcupine. The modified Wnt molecules are released from the Wnt-producing cell by seven-pass membrane protein Wntless (WLS) (Bänziger et al., 2006). It would be very informative to use a conditional knockout allele of the *Wls* gene (Carpenter et al., 2010) in combination with a suitable Cre deletor strain to study the role of Wnt signaling in the brain tissue recovery.

## Conclusion

**FIGURE 8:**
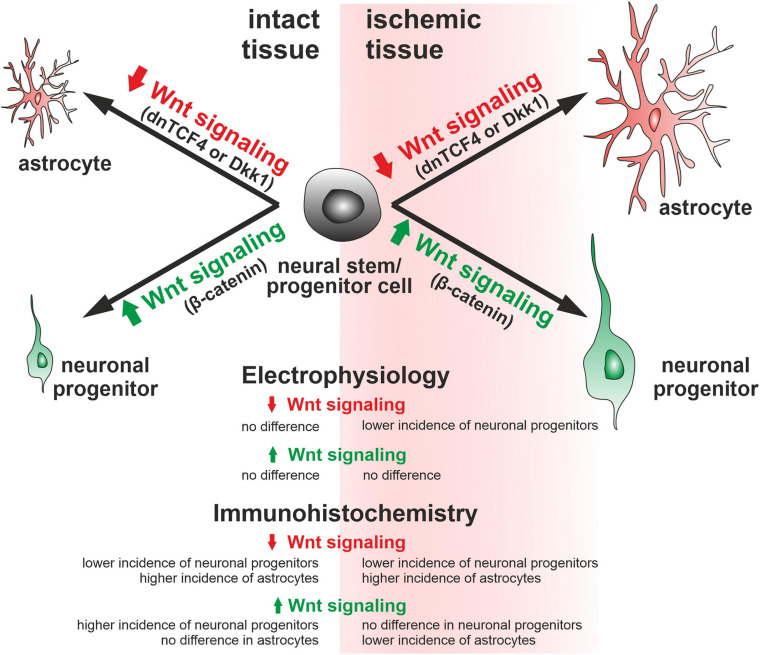
Graphical representation of the changes observed in adult mice. According to our immunohistochemical analyses, Wnt signaling inhibition (dnTCF4 or Dkk1) led to the differentiation of neural stem/progenitor cells to astrocytes, while activation of the pathway (Ex3; constitutively active β-catenin) promoted neurogenesis. A similar impact of Wnt signaling manipulation after ischemia was also confirmed by the patch-clamp technique. Larger cells represent a greater effect of Wnt signaling after ischemia. Dkk1, Dickkopf 1; dnTCF4, dominant negative T-cell factor 4; Ex3, exon 3.

In this study, we assessed the impact of the canonical Wnt pathway modulation on NS/PCs derived from the SVZ of adult mice. We observed that the Wnt pathway modulation had no effect on *in vitro* differentiated NS/PCs derived from the healthy brain. Nevertheless, when the cells were obtained from the ischemic brain, the Wnt pathway inhibition at the membrane level resulted in fewer neuron-like cells present in the cell cultures. Furthermore, the electrophysiological analysis indicated that blocking Wnt signaling affected the distribution of *K*^+^ and Na^+^ channels, and thus influenced the membrane properties of differentiated cells. The immunohistochemical analysis showed increased counts of DCX-, GFAP-, and PCNA-positive cells after the induction of FCI, while the effect of the Wnt pathway activation was more robust in the control, undamaged brain. More specifically, Wnt signaling hyper-activation increased the abundance of neuron-like DCX-positive precursors and proliferating cells, and Wnt pathway inhibition had the opposite effect (Figure 8). Our findings might help develop new strategies that ameliorate the negative effects of brain ischemia. Furthermore, it would be beneficial to determine whether active Wnt signaling might affect the course of other neurodegenerative diseases and neurological disorders, such as amyotrophic lateral sclerosis and schizophrenia.

## Data Availability Statement

The datasets generated for this study can be found in online repositories. The names of the repository/repositories and accession number(s) can be found below: https://www.ebi.ac.uk/arrayexpress/experiments/E-MTAB-9935/.

## Ethics Statement

The animal study was reviewed and approved by Animal Care Committee of the Institute of Experimental Medicine, Czech Academy of Sciences (approval numbers 18/2011, 146/2013, and 2/2017).

## Author Contributions

MA and VK designed the experiments and supervised the project. MA, VK, JK, and LJ coordinated the experiments. JK was responsible for the tissue isolation and cell culture preparation. JK, PH, TK, DD, DKo, and OB contributed to the electrophysiological studies. JK, LJ, and OB acquired and analyzed the data from the immunochemical experiments. MC created and described the script for cell counting. DKi and PH performed the MCAO. MV carried out the Western blotting experiments. LJ performed and analyzed the scRNA-seq and the RT-qPCR experiments. LJ and VK supervised the breeding of the transgenic mouse strains. MT and ZK provided the *Catnb*^*lox(ex3)*^ mice. JK and LJ wrote the manuscript. MA, VK, and TK revised the manuscript. All authors read and approved the final version of the manuscript.

## Conflict of Interest

The authors declare that the research was conducted in the absence of any commercial or financial relationships that could be construed as a potential conflict of interest.

## Abbreviations

4OHT: (Z)-4-hydroxytamoxifen
A488: Alexa Fluor hydrazide 488
A594/660: Alexa Fluor 594 or 660
aCSF: artificial cerebrospinal fluid
APC: adenomatous polyposis coli
B27: B27 supplement
bFGF: fibroblast growth factor-basic
BMP: bone morphogenetic protein
cDNA: complementary deoxyribonucleic acid
*C*_*M*_: membrane capacitance
CNS: central nervous system
CO: corn oil
CO_2_: carbon dioxide
COPs: committed oligodendrocyte precursors
Com: complex current pattern
*C*_*t*_: cycle threshold
*Ctnnb1*: gene encoding β -catenin
CTRL: control, non-operated mice
Cy3: cyanine dye 3
DAPI: 4 ′, 6-diamidino-2-phenylindole
DCX: doublecortin
Dkk1: Dickkopf 1
Dn: dominant negative
DNase: deoxyribonuclease
E2-3: exons 2–3
EGF: epidermal growth factor
EGFP: enhanced green fluorescent protein
EGFR: epidermal growth factor receptor
EGTA: ethylene glycol-bis(β -aminoethyl ether)-N,N,N ′, N ′ -tetraacetic acid
ERT2: mutant form of estrogen receptor
EtOH: ethanol
Ex3: exon 3
FACS: fluorescence-activated cell sorting
FCI: focal cerebral ischemia
FP: forward primer
*GAPDH*: *glyceraldehyde-3-phosphate dehydrogenase*
GFAP: glial fibrillary acidic protein
GSK-3 β: glycogen synthase kinase 3 β
HEPES: 4-(2-hydroxyethyl)-1-piperazineethanesulfonic acid
i.p.: intraperitoneal
IgG: immunoglobulin G
IR: input resistance
*K*^+^: potassium
*K*_*A*_: fast activating and inactivating outwardly rectifying *K*^+^ current
*K*_*A*_/*C*_*M*_: current density of *K*_*A*_
*K*_*DR*_: delayed outwardly rectifying *K*^+^ current
*K*_*DR*_/*C*_*M*_: current density of *K*_*DR*_
*K*_*IR*_: inwardly rectifying *K*^+^ current
*K*_*IR*_/*C*_*M*_: current density of *K*_*IR*_
LRP: low-density lipoprotein receptor-related protein
LV: lateral ventricle
MAP2: microtubule-associated protein 2
MCAO: middle cerebral artery occlusion
mRNA: messenger ribonucleic acid
n: number of specimens/cells
Na^+^: sodium
Na^+^/*C*_*M*_: current density of Na^+^ channels
NG2: neuron-glial antigen 2
*Nkd1*: *naked cuticle homolog 1*
NMDG: *N*-Methyl -D-glucamine
NS/PCs: neural stem/progenitor cells
OPCs: oligodendrocyte precursor cells
Out: outwardly rectifying current pattern
pA: polyadenylation site
pas: passive current pattern
PB: phosphate buffer
PBS: phosphate-buffered saline
PCNA: proliferating cell nuclear antigen
PDGFR α: platelet-derived growth factor receptor alpha
PFA: paraformaldehyde
PGK-Neo: neomycin resistance cassette
PLL: poly-L-lysine
PTB: pentobarbital
RP: reverse primer
RT: room temperature
RT-qPCR: reverse transcription quantitative polymerase chain reaction
S.D.: standard deviation
S.E.M.: standard error of the mean
SA: splice acceptor site
scRNA-seq: single-cell RNA sequencing
SGZ: subgranular zone
SVZ: subventricular zone
TAM: tamoxifen
TCF/LEF: T-cell factor/lymphoid enhancer factor
Td: tandem dimmer
*Tnfrsf19*: *tumor necrosis factor receptor superfamily, member 19*
*Troy*: *tumor necrosis factor receptor superfamily, member 19*
TTX: tetrodotoxin
*V*_*M*_: membrane potential
WLS: Wntless
Wnt: Wingless/Integrated
WT: wild-type.
