# Supplementary figures and images for: Wnt/β-Catenin Signaling Promotes Differentiation of Ischemia-Activated Adult Neural Stem/Progenitor Cells to Neuronal Precursors
Source: Front Neurosci. 2021 Feb 25;15:628983. doi: 10.3389/fnins.2021.628983 (PMC7947698; doi:10.3389/fnins.2021.628983)

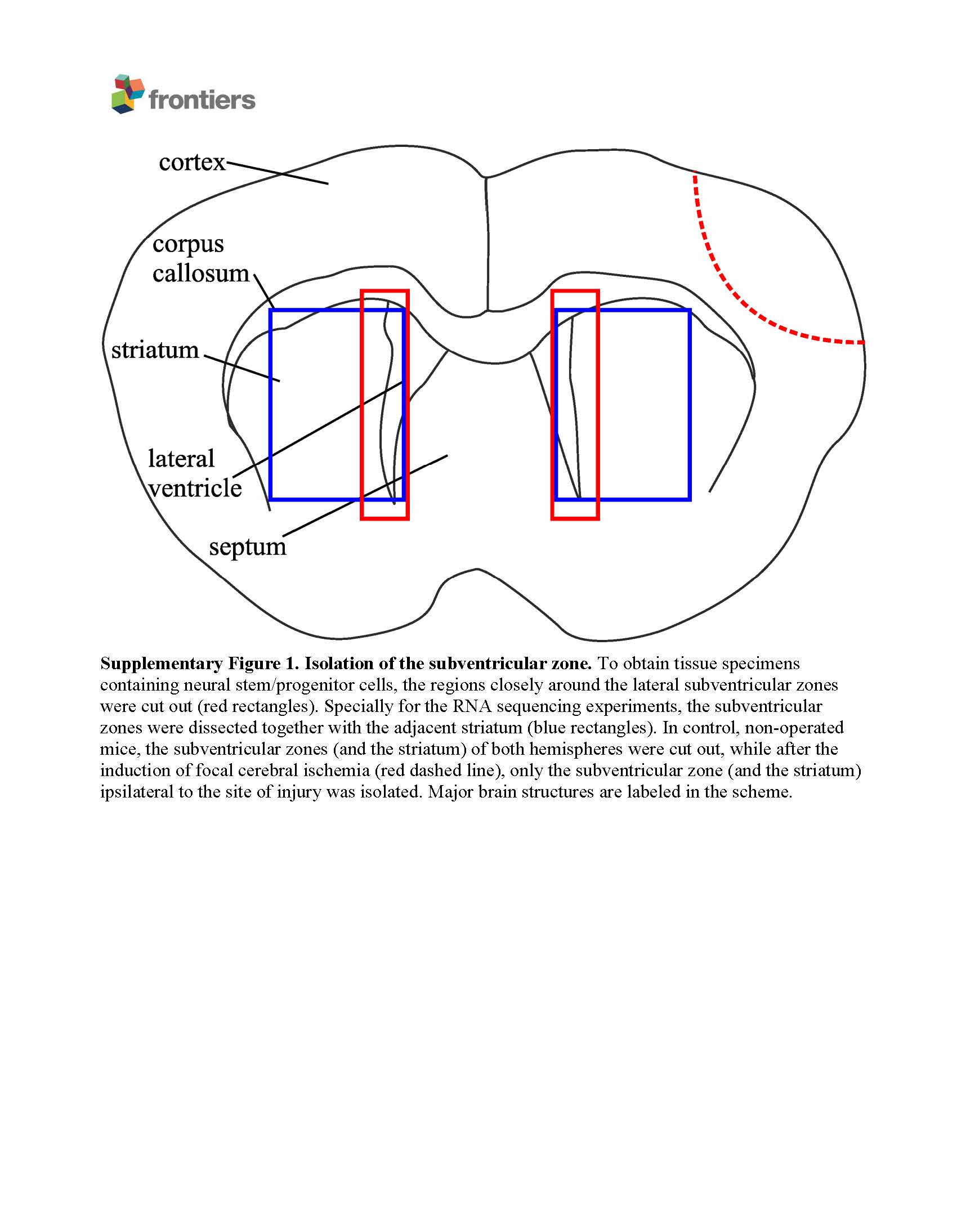

Supplement: Supplementary file 1 [file Image_1.jpg]

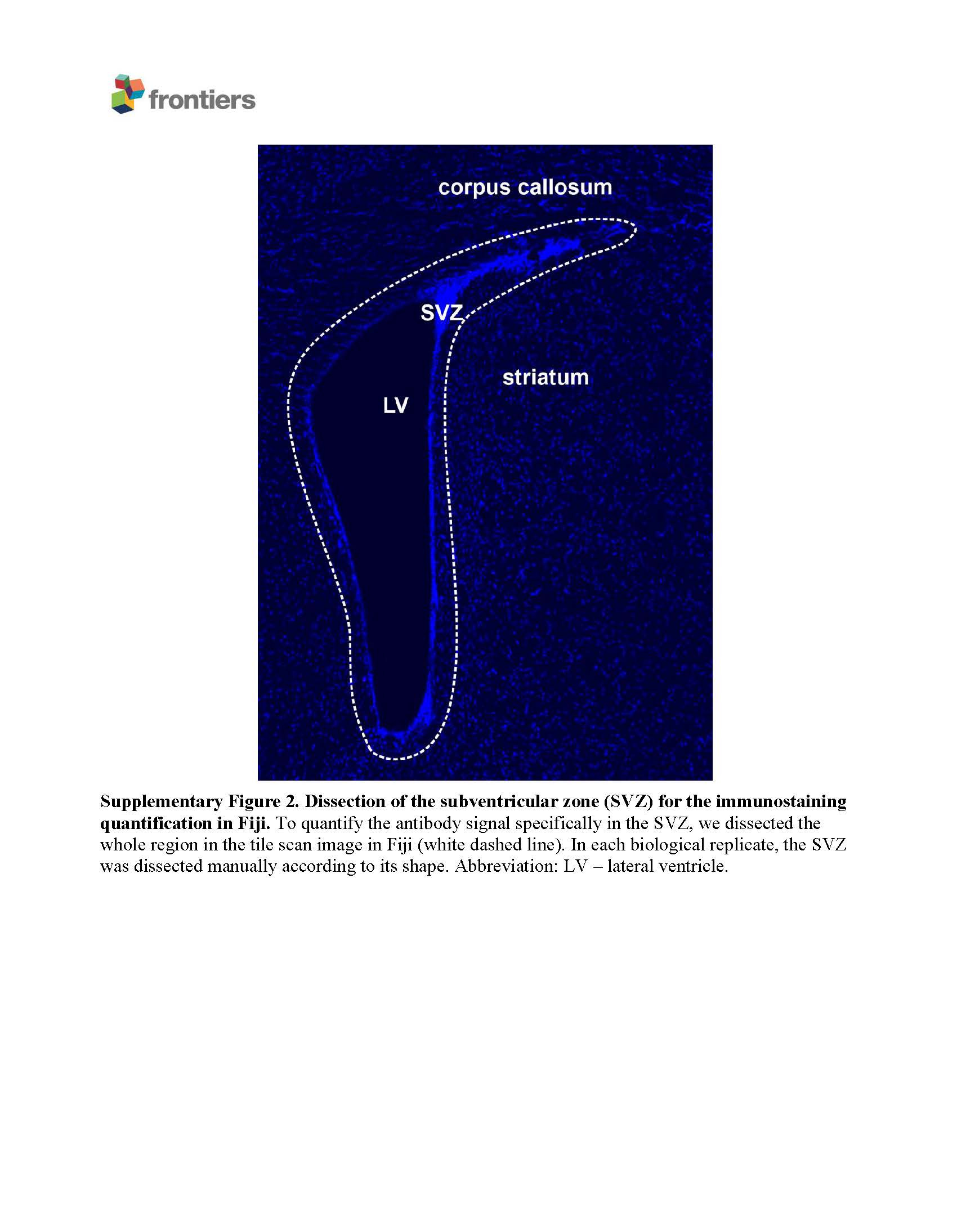

Supplement: Supplementary file 2 [file Image_2.jpg]

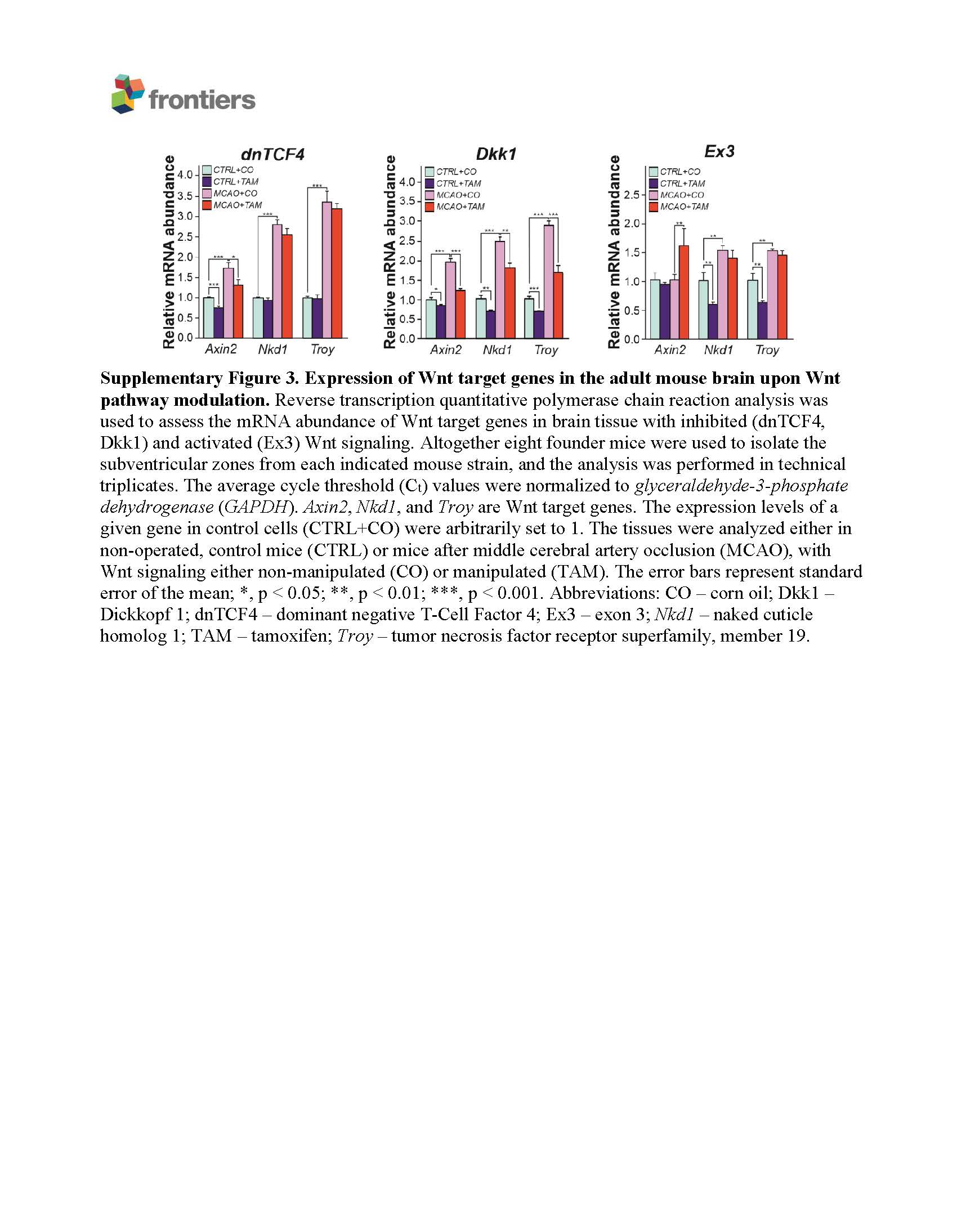

Supplement: Supplementary file 3 [file Image_3.jpg]

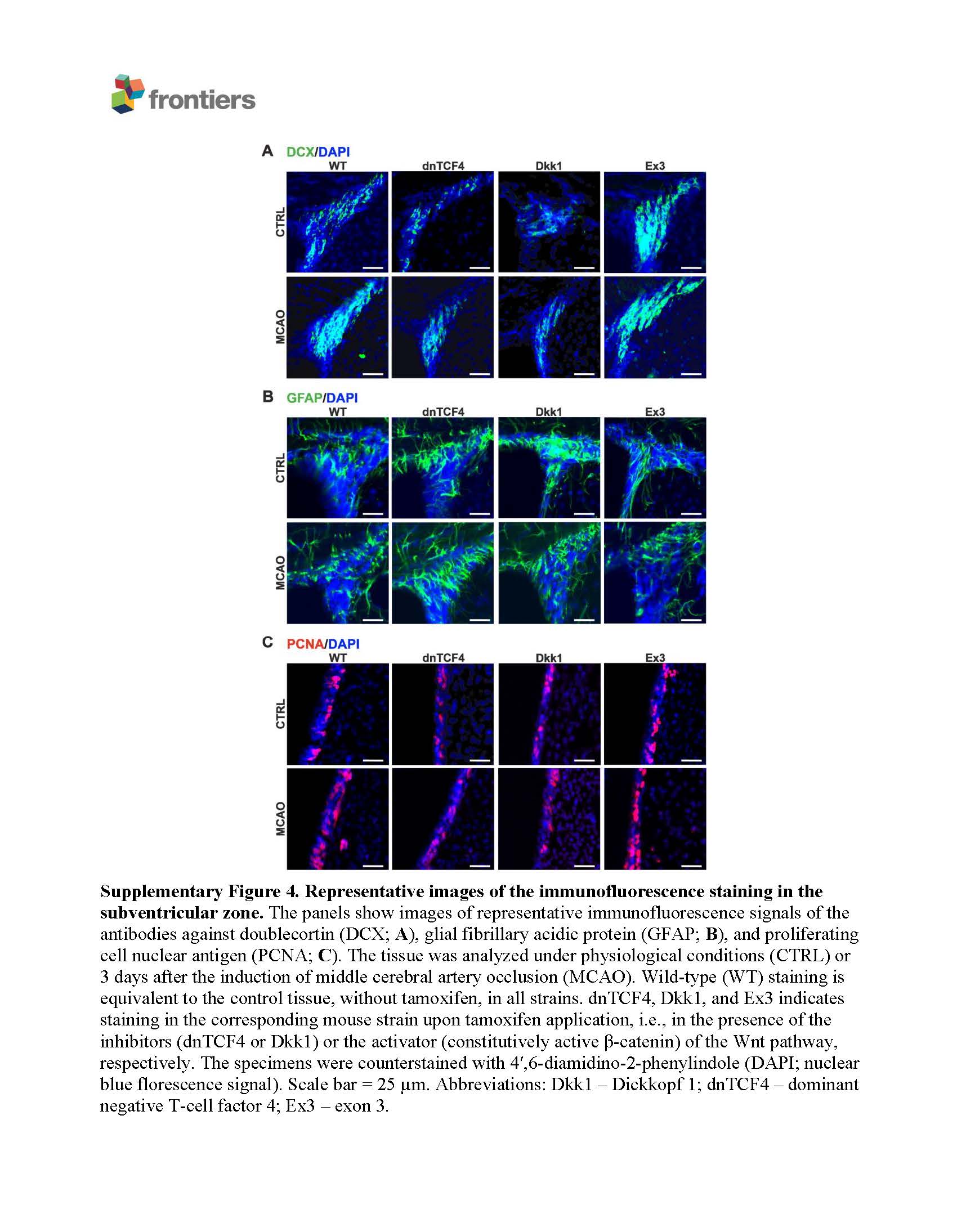

Supplement: Supplementary file 4 [file Image_4.jpg]
